# *De novo* characterization of *Phenacoccus solenopsis* transcriptome and analysis of gene expression profiling during development and hormone biosynthesis

**DOI:** 10.1038/s41598-018-25845-3

**Published:** 2018-05-15

**Authors:** Surjeet Kumar Arya, Yogeshwar Vikram Dhar, Santosh Kumar Upadhyay, Mehar Hasan Asif, Praveen Chandra Verma

**Affiliations:** 1 CSIR-National Botanical Research Institute, (Council of Scientific and Industrial Research) Rana Pratap Marg, Lucknow, UP-226001 India; 2 Academy of Scientific and Innovative Research (AcSIR), Anusandhan Bhawan, Room No: 310, 2-Rafi Marg, New Delhi, India; 3 Department of Botany, Panjab University, Chandigarh, 160014 India

## Abstract

The cotton mealybug *Phenacoccus solenopsis* is a devastating pest of cotton causing tremendous loss in the yield of crops each year. Widespread physiological and biological studies on *P. solenopsis* have been carried out, but the lack of genetic information has constrained our understanding of the molecular mechanisms behind its growth and development. To understand and characterize the different developmental stages, RNA-Seq platform was used to execute *de-novo* transcriptome assembly and differential gene expression profiling for the eggs, first, second, third instar and adult female stages. About 182.67 million reads were assembled into 93,781 unigenes with an average length of 871.4 bp and an N50 length of 1899 bp. These unigenes sequences were annotated and classified by performing NCBI non-redundant (Nr) database, Kyoto Encyclopedia of Genes and Genomes (KEGG) and Clusters of Orthologous Groups (COG), Gene ontology (GO), the Swiss-Prot protein database (Swiss-Prot), and nearest related organism *Acyrthosiphon pisum* (pea aphid) database. To get more information regarding the process of metamorphosis, we performed a pairwise comparison of four developmental stages and obtained 29,415 differentially expressed genes. Some of the differentially expressed genes were associated with functional protein synthesis, anti-microbial protection, development and hormone biosynthesis. Functional pathway enrichment analysis of differentially expressed genes showed the positive correlation with specific physiological activities of each stage, and these results were confirmed by qRT-PCR experiments. This study gives a valuable genomics resource of *P. solenopsis* covering all its developmental stages and will promote future studies on biological processes at the molecular level.

## Introduction

The cotton mealybug, *Phenacoccus solenopsis* (Pseudococcidae) is a genetically diverse and highly destructive invasive insect pest of many horticultural and agricultural crops, worldwide^1^. It infests more than 200 different plant species and causes significant yield loss^2–6^. It affects the plants by feeding and vectoring debilitating plant viruses, as well^7^. It feeds phloem-sap and promotes the growth of damaging fungi on honeydew excretions deposited on plant parts^5^. Annual worldwide crop loss is estimated around hundreds of millions of dollars^5^. Other mealybug species infesting cotton in India include *Maconellicoccus hirsutus* (pink hibiscus mealybug), *Paracoccus marginatus* Williams and Ganara de willink (Papaya mealybug) and *Nipaecoccus viridis* (spherical mealybug)^5^. However, the cotton mealybug *P. solenopsis* is the most destructive species, which affects 65% of cotton producing area in India^8–10^. Due to the lack of efficient mealybug resistance germplasm, the complexity of plant-mealybug interactions got increased along with the development of resistant pest biotypes and outbreaks of mealybug causing substantial crop losses are being regularly reported^11–13^. Cotton breeders and seed plant growers are in continuous efforts to find an efficient genetic strategy for their control in cotton plants.

Hemi-metabolous insects, such as aphids, whiteflies, leafhoppers and true bugs undergoes morphogenesis as similar to that seen in the larva-to-pupa and pupa-to-adult transitions of holo-metabolous insects, to produce mature external wings and genitalia. However, the change of one form to another is not prominent because crawler or nymphs look similar in appearance to their adult form. In spite of all these differences in metamorphosis, both holo-metabolous and hemi-metabolous process are regulated by general mechanisms involving molting steroid 20-hydroxyecdysone (20E) and the sesquiterpenoid, juvenile hormone (JH)^14–16^. JH regulates many processes of insect physiology, such as reproduction, development, diapauses, and metamorphosis^16–18^. Subsequently, regulation of JH levels is crucial throughout an insect’s life.

The transcriptome sequencing data can be used to infer the information related to the development and other metabolic processes in an organism. In insect, growth, molting and other regulatory processes are mostly regulated by the hormonal balance between acyclic sesquiterpenoid produced at the corpus allatum (CA), and ecdysteroid synthesized at the prothoracic glands^19^. The family of sesquiterpenoid primarily limited to insects^20^. Eight different types of JHs have been identified in insects^21^. They are synthesized through the mevalonate pathway (MVAP) and involve thirteen discrete enzymatic reactions. The synthesis of JH is conventionally divided into two steps, early (MVAP) and late (JH branch) steps. In early steps, MVAP has to form FPP through a series of sequential steps that involves the critical roles of major and minor enzymatic reactions. Mevalonate first transformed into isopentenyl diphosphate (IPP) in the presence of three enzymatic steps. FPP synthase (fpps), a short-chain prenyltransferase, generates a FPP by completing two sequential couplings reactions. In the first reaction, IPP and dimethylallyl pyrophosphate (DMAPP) condense in a head to tail manner to produce geranyl diphosphate (GPP). Then, condensation reaction occurs repeatedly where GPP reacts with IPP and yields FPP. Many FPP synthases have been identified in several insects and typically active as homodimers^22,23^. The late steps of JHs biosynthesis involve the conversion of FPP to farnesol (FOL) that is catalyzed by an FPP phosphatase (fppase or fppp). Afterward, farnesol undergoes two sequential oxidation reactions that generate farnesal and farnesoic acid (FA). Further, biosynthetic step involves methyl esterification and epoxidation which are catalyzed by an acid methyltransferase (Jhamt) and an epoxidase (epox). The last step of the biosynthetic reaction is usually considered to be JH-specific^21,24^.

The steroid hormone ecdysone has roles in insect molting and metamorphosis through its timely release into the circulating hemolymph from the prothoracic gland^19,24^. In case of ecdysteroid, phylum arthropoda lacks the squalene synthase enzyme, and its synthesis relied on the continuous supply of external sterol source derived from the diet. It usually requires dealkylation and a series of hydroxylation steps^19^. Previous studies reported that the most active form of ecdysone is 20-hydroxyecdysone (20E) which binds effectively to the nuclear receptor (EcR) of the target tissue to elicit specific changes in the transcription of genes. The complete biosynthesis of 20E from cholesterol is catalyzed by cytochrome P450 enzymes encoded by the halloween family of genes. These families of genes include: phantom/Cyp306A1 (phm), spook/Cyp307A1 (spo), spookier/Cyp307A2 (spok), shadow/Cyp315A1 (sad)^24–27^.

In this study, we analyzed the high throughput RNA sequencing data (RNA-Seq) to establish transcriptome profile of four developmental stages of *P. solenopsis*, enabling the identification of potential target genes based on differential expression. We generated over 100 million bases of high-quality RNA sequence using Illumina technology, which was assembled into 93,781 distinct sequences. RNA-Seq data from various developmental stages enabled identification of vital differentially expressed genes (DEGs) involved in the development and various biosynthetic pathways. Those essential genes may also be a potential target for the control of mealybug in plants through RNA interference (RNAi).

## Materials and Methods

### Insect rearing

*P. Solenopsis* was reared on cotton plants at 25–28 °C, 70–80% relative humidity and photoperiods of 16:8 (L: D) h for biological studies in the laboratory at CSIR-National Botanical Research Institute, Lucknow, India, as described earlier^28^. Egg, first instar, second instar and third instar nymphs, and female adults) of *P. solenopsis* were collected separately from the reared population. Due to the limited number of biological samples, egg and first instar nymphs were pooled and considered as a single sample as EggI, while second instar, third instar and female adults were taken, separately.

### Construction of cDNA library, Illumina sequencing and assembly

Total RNA from above samples were extracted separately using TRIzol reagent (Sigma Aldrich, USA) following the manufacturer’s instructions. DNA contaminants were removed using DNase enzyme (Invitrogen) digestion, followed by rRNA removal using Ribo-Minus (Invitrogen). The RNA samples were quantified using a NanoDrop ND-1000 spectrophotometer (NanoDrop Technologies, USA) and qualified by Agilent Bioanalyzer (Agilent Technologies, USA). The processed RNAs with RNA integrity number (RIN) values (≥7.00) were sent for sequencing using Illumina HiSeq. 2500 platform (2 × 100 bp strategy). The cDNA libraries were constructed according to Illumina TruSeq™ Stranded mRNA sample preparation guide for Illumina Paired-End sequencing service provided by Scigenome, India. Raw reads generated through HiSeq. 2500 were filtered to exclude low quality reads. The transcriptome data of *P. Solenopsis* was processed through a filtering process while considering the Q-value of 30 using the NGS QC Toolkit^29^. The clean reads from four cDNA libraries were pooled and subjected to *de-novo* assembly using Trinity software^30^ with default parameters (Supplementary Fig. S1). High-quality reads had a Phred score over 30 across more than 70% of the bases.

### Functional annotation and differential gene expression analysis

Assembled unigenes were annotated by a BLASTx search (E-value < 10−5) against the NCBI non-redundant (Nr) database (ftp://ftp.ncbi.nih.gov/blast/db/), Clusters of Orthologous Groups (COG) (http://www.ncbi.nlm.nih.gov/COG/) and Kyoto Encyclopedia of Genes and Genomes (KEGG) (http://www.genome.jp/kegg/), Gene ontology (GO) (http://www.geneontology.org/), the Swiss-Prot protein database (Swiss-Prot) (http://www.uniprot.org/), and nearest related organism *Acyrthosiphon pisum* (pea-aphid)^31^. AgriGO (http://bioinfo.cau.edu.cn/agriGO/) online tool was used for GO mapping of the annotated contigs. The gene expression abundance was calculated using RSEM^32^ and compared using edgeR software^33^. The differentially expressed genes were distinguished on the basis of stage-specific genes with their log_2_ Fold change value in the respective developmental stages (if P < 0.05, |log2| ≥ 2 and FDR < 0.05 to be considered statistically significant).

### Pathway enrichment analysis of the differentially expressed genes (DEGs)

To obtain a comprehensive perspective on the molecular basis of development, we focused on differential genes that have a role in hormone biosynthesis pathway and development. The enrichment analysis of GO and KEGG pathways was performed with DEGs by using KOBAS 3.0 software [http://kobas.cbi.pku.edu.cn/home.do]^34,35^. Pathway Enrichment analysis finds the most significantly enriched pathways by applying a hypergeometric test to map all DEGs to give terms in the GO database by comparing it with the whole genome background. The mapped enriched pathways include metabolic or signal transduction. The formula used for the calculation was:

$${\rm{P}}=1-\sum _{{\rm{i}}=0}^{{\rm{M}}-1}\frac{(\begin{array}{c}{\rm{m}}\\ {\rm{i}}\end{array})\,(\frac{{\rm{N}}-{\rm{M}}}{{\rm{n}}-{\rm{i}}})}{({\rm{N}}/{\rm{n}})}$$

here, N represents the number of all genes that with KEGG annotation, n is the number of DEGs in N, M is the number of all genes annotated to specific pathways, and m is the number of DEGs in M. The pathway with Q value 0.05 is defined as the enriched one. For the Detailed method one can refer to the earlier reports^36,37^. Visualization of data was accomplished using the JMP 13.0 software^38^.

### qRT-PCR analyses

The expression of 10 selected genes during developmental stages in *P. solenopsis* was validated using qRT-PCR. The gene-specific primers were designed using Primer 3.0 program (Table S1). The qRT-PCR reactions were performed on 7500 Real-Time PCR Systems (Applied Biosystems, USA) using the following program cycles with step involves: an initial denaturation step of 20 s at 50 °C, 95 °C for 10 min., and 40 cycles of 15 s at 95 °C, and 1 min at 60 °C. Reaction was followed by melt curve analysis at default parameters to check the PCR specificity by steady increase in temperature from 60 °C to 90 °C. The expression level was estimated using the 2^−ΔΔCT^ method and normalized with three selected reference genes (*β-tubulin*, *α-tubulin*, and *rpl32*) based on expression studies in different development stages of *P. solenopsis*^28^. Values for each developmental stage were taken as an average of three technical replicates of each biological replicate. Statistical analysis was done with using SPSS 16.0.

### Data Accessibility

The transcriptomic dataset for different developmental stages of *Phenacoccus solenopsis* has been in deposited in the NCBI SRA database, under the SRA accession number of SRP133470.

## Results

### Whole-transcriptome sequencing and data assembly

A total of 56.11, 45.80, 48.40 and 32.36 million raw-reads were generated from EggI, second instar, third instar and adult females respectively. After removing low quality and adaptor reads, 50.62, 40.04, 40.29 and 29.70 million clean-reads were obtained for each. The base sequence of the total data was approximately 7.27, 11.6, 10.98 and 13.74 gigabases, respectively. The entire data were deposited in the NCBI SRA database, under the SRA accession number of SRP133470. These reads were assembled into 93,781 distinct contigs with an average length of 871 bp and N50 length of 1899 bp (Tables 1 and 2).

**Table 1:** Summary of statistical data for the transcriptomes of *P. solenopsis* developmental stages.

Sample name	Raw reads	Clean reads	% of HQ reads	Q > 30 (%)	GC content (%)
**A-**R1	28055790	25311685	98.6	90.23	39.72
**A-**R2	28055790	25311685	96.9	84.35	39.48
**B-**R1	22904667	20021109	98.42	88.88	40.12
**B-**R2	22904667	20021109	96.25	80.13	39.7
**C-**R1	24200312	20147343	98.12	88.12	39.23
**C-**R2	24200312	20147343	95.31	74.78	38.77
**D-**R1	16183365	14853656	91.78	71.23	40.12
**D-**R2	16183365	14853656	91.78	65.34	39.95

**Table 2:** Summary of assembly statistics for the different developmental stages of *P. solenopsis*. N50 is the 50% length of all unigenes.

Number of genes	GC percentage	N50	Average contig lengths	Total transcripts assembled
93,781	35.69	1899	871.4	1,11,871

### Gene Annotation

**Figure 1:**
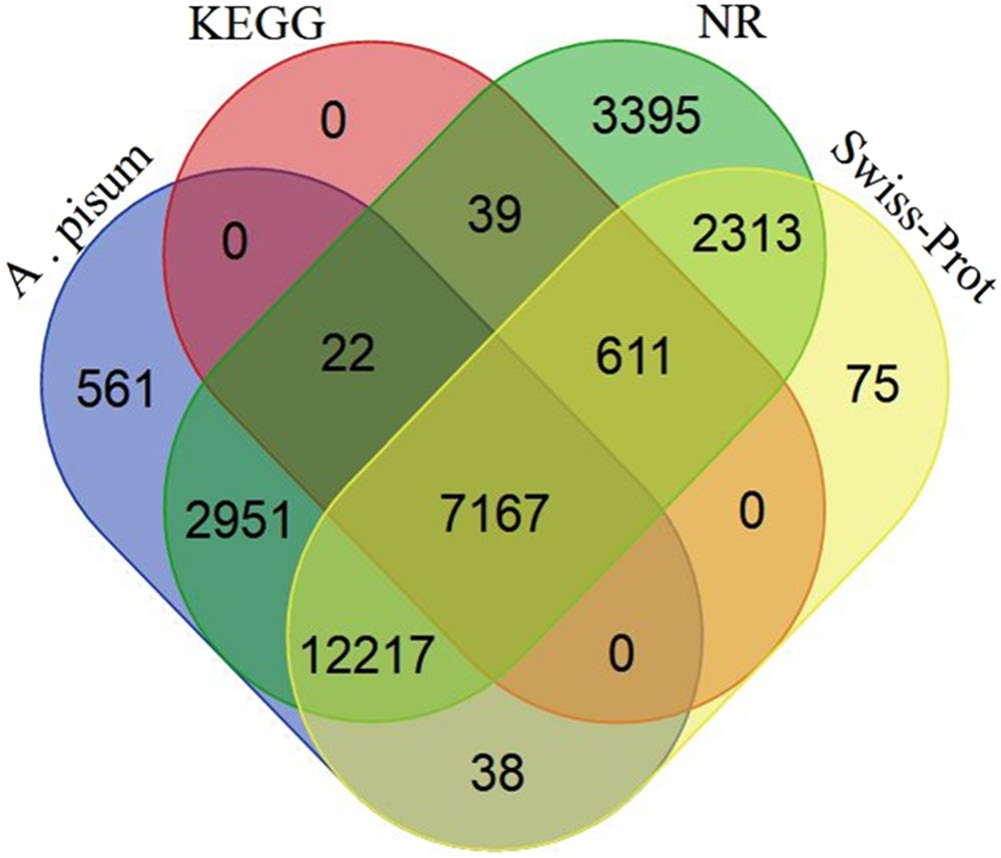
A Venn diagram illustrating shared and unique DEGs annotated in nr, Swissprot, COG, GO and KEGG public databases. Among 33,831 DGEs, 29,415 annotated in at least one of the public databases, including in nr, Swiss-prot, COG KEGG and GO databases, respectively.

**Figure 2:**
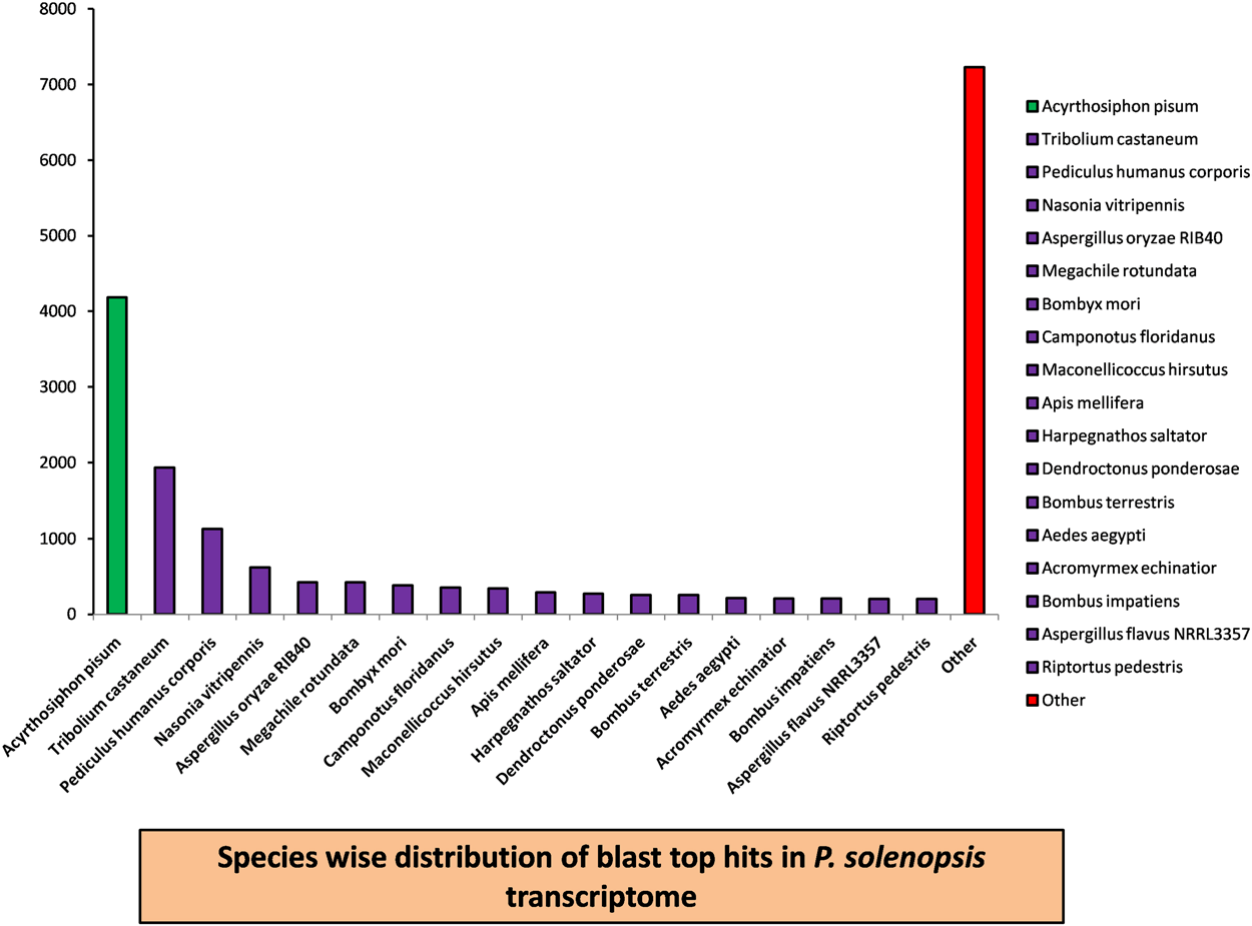
The top-eighteen species distribution of the BLASTX results. Unigenes were aligned with the NCBI-Nr protein database with a cutoff E value < 10−5. Different colors represent different species.

A total of 38,725 contigs were mapped to NR, 31965 to Swiss-prot, 16,095 to COG, 7,839 to KEGG, and 31904 to *A. pisum* databases (Fig. 1). Overall, a total of 38,725 unique contigs were annotated (Table 3). A total of 11,896 contigs were annotated by a BLAST hit with 18 insect species (Fig. 2). As expected, the top hits were found in the insect genomes, especially hempiteran and lepidopteran. *A. pisum* and *Tribolium castaneum* were the first and second top-hit species with the annotations of 4,182 and 1,933 contigs followed by *Pediculus humanus corporis*, *Nasonia vitripennis*, and *Aspergillus oryzae* RIB40 (Table S2).

**Table 3:** Functional annotations of total unigenes in *P. solenopsis*. Nr: non-redundant database; Swiss-Prot: Swiss Protein database; KOG: Clusters of Orthologous Groups of proteins; KEGG: Kyoto Encyclopedia of Genes and Genomes; *A. pisum*.

Sequence File	NR	Swiss-prot	COG	KEGG	*A. pisum*	Total
Unigenes	38,725	31,965	16,095	7,839	31,904	29,415

### GO mapping and pathway analysis

**Figure 3:**
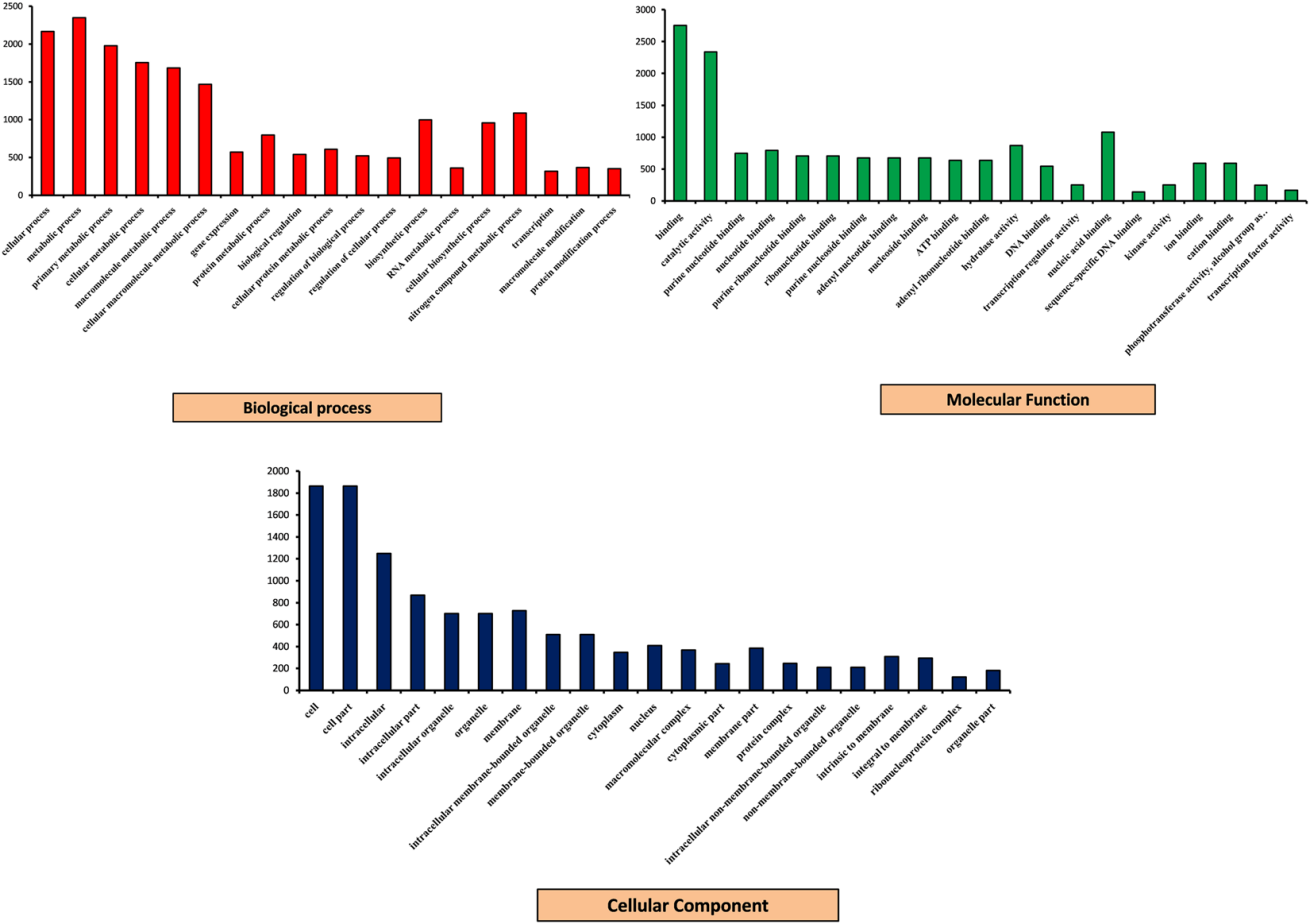
GO unigene categories. The unigenes were annotated in three main categories: biological process (blue), cellular component (red) and molecular function (green). The left side of the y-axis represents the percentage of a specific category of genes for a main category. The right side of the y-axis represents the number of genes in a category.

The GO mapping was performed using *A. pisum* data available at AgriGO database. A total of 102 biological processes, 68 molecular functions, and 27 cellular components were mapped. Cellular process (2167 genes), metabolic process (2348 genes), primary metabolic process (1977 genes), cellular metabolic process (1756 genes), macromolecule metabolic process (1685 genes), nitrogen compound metabolic process (1087 genes), biosynthetic process (999 genes), nucleobase, nucleoside, nucleotide and nucleic acid metabolism (994 genes) were the major biological processes (Fig. 3, Tables S3–S5). In the KEGG assignment, 7839 unigenes were mapped to 259 different pathways (Supplementary excel file 1). Out of them, 20.16% pathways were classified into the pathway related to metabolism, with most of them involved in oxidative phosphorylation (27 unigenes), purine and pyrimidine metabolism (104 unigenes), carbon metabolism (154 unigenes), biosynthesis of amino acids (37 unigenes), Wnt signaling pathway (78 unigenes), and FoxO signaling pathway (69 unigenes) (Supplementary excel file 2).

### Differential gene expression analyses among *P. solenopsis* developmental stages

To analyze the differential expression levels of genes between the major developmental stages, up and down-regulated genes were estimated between each pair of *P. solenopsis* such as EggI vs. second instar, second vs. third instar, third instar vs. adult female, adult female vs. EggI. Genes with two-fold variation in expression with statistical significance between two or more developmental stages were considered as differentially expressed. The expressions profile of the differential gene was changed as the insect move to the different instar of the stages. The top 10 genes for each comparison are listed in Table S6. We then performed four comparisons viz. EggI vs. second, second vs. third and third vs. adult female, and adult female vs EggI and identified the genes showing significant variation in expression.

**Figure 4:**
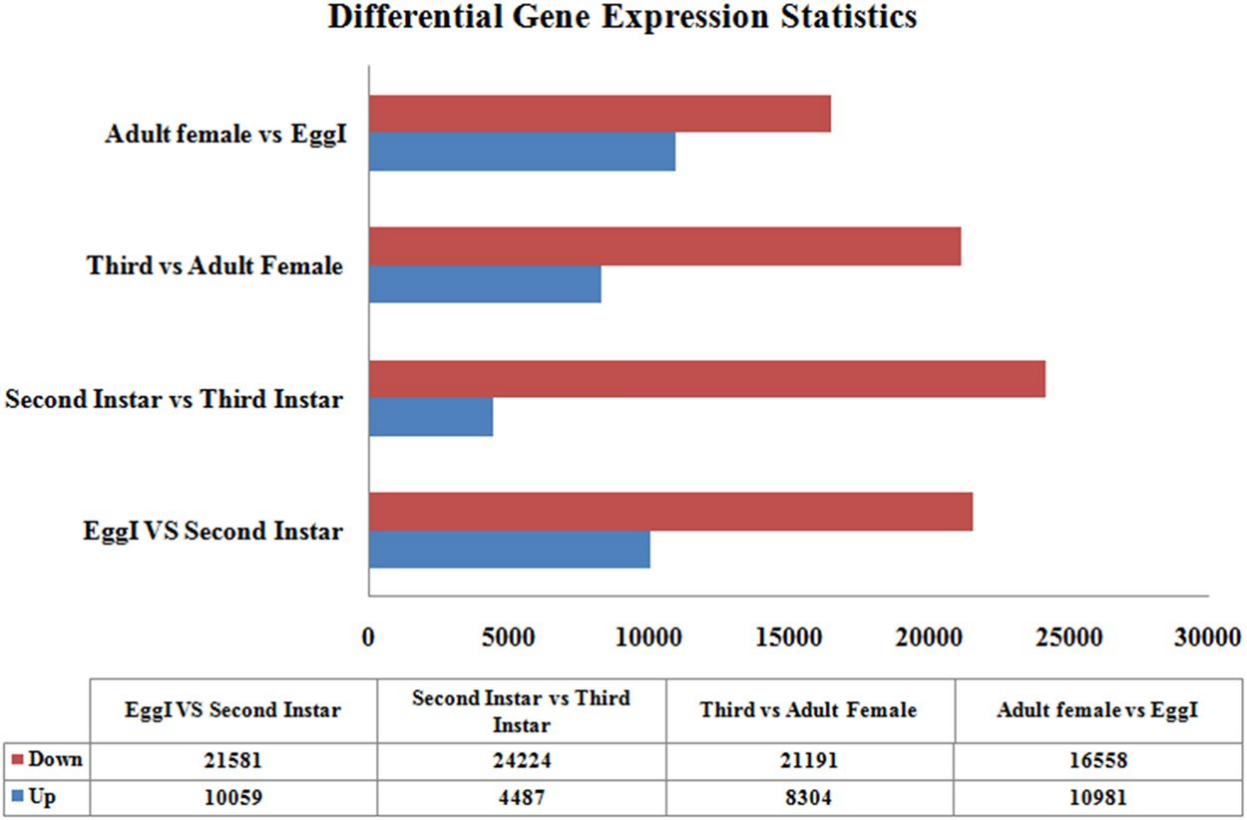
Comparison of the numbers of unigenes in different developmental stages in *P. solenopsis*. Up-regulated unigenes are marked in red, and down-regulated unigenes are marked in green.

**Figure 5:**
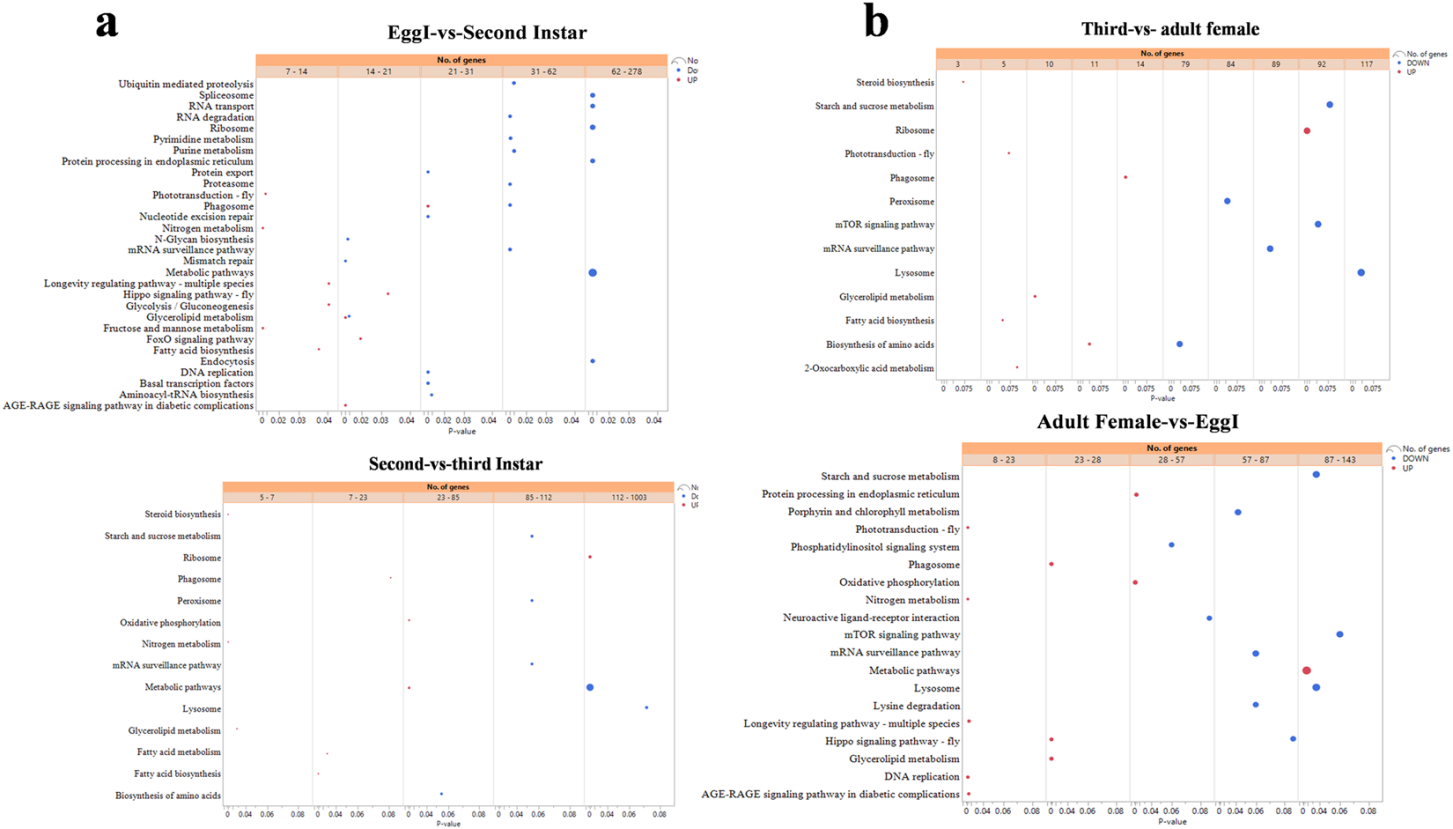
Enrichment analysis of KEGG pathways in comparisons of different stages. The x-axis indicates the p value calculated in enrichment test. Numbers of genes that were up-regulated (red) or down-regulated (blue) in comparisons of (**a**) EggI vs. Second instar and Second vs. third instar, (**b**) Third vs. adult and Adult female vs. EggI are shown. The size of circles indicates the number of genes in that pathway. Red circles represented up-regulated genes, while blue circles represented down-regulated genes.

In total, 31,640 deferent genes showed significant expression change in hatching-to-second instar process, including 10,059 up-regulated and 21,581 down-regulated genes (Fig. 4). Most of the up-regulated genes in the second instar categorized into the cellular process, metabolic process, organic substance metabolic process, and biosynthetic process (Supplementary excel file 3). Other categories, such as organic cyclic compound biosynthetic process (biological process categories), membrane-bound organelle (cellular component categories), hydrolase activity, oxido-reductase activity, etc. were also enriched. On the other hand, the high expressing genes in the egg stage were mainly enriched for DNA integration, protein folding, and protein synthesis processes such as ATP binding and ribosome. In down-regulated genes, the representative abundant enrichment GO terms included developmental process, biosynthetic process, cell differentiation, and biological regulation, intracellular membrane-bound organelle, (cellular component ontology), nucleotide acid binding, DNA binding, transcription factor activity, transcriptional regulation activity (molecular function ontology). Furthermore, we also performed KEGG enrichment analysis (Fig. 5a, Supplementary excel 4 and Table S7). FoxO signaling pathway, Hippo signaling pathway, fructose and mannose metabolism, glycerolipid metabolism, nitrogen metabolism, and glycolysis/gluconeogenesis were up-regulated, and glycerolipid metabolism, N-Glycan biosynthesis, and protein export were down-regulated.

A total of 28,711 significantly expressed genes (including 4,487 up-regulated genes and 24,224 down-regulated genes) were obtained in the third instar vs. second instar library (Fig. 4). According to GO enrichment analysis (Supplementary excel file 3), when second instar mealybug molt to the third instar many metabolic process genes were found to be up-regulated and categorized into RNA catabolic process, ribosome biogenesis, cellular nitrogen compound catabolic process, organic cyclic compound catalytic process, etc. For the down-regulated genes, the representative abundant GO terms included developmental process, biosynthetic process, cell differentiation, and biological regulation, intracellular membrane bound organelle, (cellular component ontology), nucleotide acid binding, DNA binding, transcription factor activity, transcriptional regulation activity (molecular function ontology). In the KEGG enrichment analysis (Fig. 5a, Supplementary excel file 4 and Table S7), the three up-regulated pathways include oxidative phosphorylation, ribosome, and fatty acid biosynthesis. The down-regulated genes were primarily involved in biosynthesis of amino acids and starch and sucrose metabolism.

A comparison between third instar and adult female revealed 29,495 significantly differentially expressed genes, including 8,304 up-regulated genes and 21,191 down-regulated genes (Fig. 4). According to GO enrichment analysis (Supplementary excel file 3), when second instar molt to the third instar many metabolic process genes were found to be up-regulated and categorized into cellular process, biosynthetic process, single-organism process, macromolecular process, ribonucleoprotein complex (cellular component categories), structural molecule activity (molecular function categories). For the down-regulated genes, the abundant enrichment GO terms included biological regulation, primary metabolic process (biological process ontology), membrane-bounded vesicle, (cellular component ontology), transport, protein binding (molecular function ontology). In the KEGG analysis (Fig. 5b, Supplementary excel file 4 and Table S7), the three up-regulated pathways include phagosome, biosynthesis of amino acids, glycerolipid metabolism, ribosome, photo-transduction, and 2-oxocarboxylic acid metabolism. The down-regulated genes were primarily involved in mTOR signaling pathway and starch and sucrose metabolism.

When differential gene expression was analyzed between the adult female and EggI transcriptome, 27,539 genes exhibited significantly differential expression. Of these genes, 10,981 were up-regulated, and 16,558 were down-regulated in the adult transcriptome (Fig. 4). Based on GO enrichment analysis (Supplementary excel file 3), most of the up-regulated genes in the adult female categorized into the metabolic process, protein metabolic process and cellular biosynthetic process. Other categories, such as gene expression, cellular process (biological process ontology), ribosomal subunit (cellular component ontology), and structural molecule activity, nucleic acid binding and organic cyclic compound binding (molecular function ontology) were also enriched. On the other part, the high expressing genes in the egg stage were mainly enriched for DNA integration, protein folding, and protein synthesis process such as ribonucleoprotein complex, ATP binding, ribosome. For the down-regulated genes, the representative abundant enrichment GO terms included cell cycle process, cell differentiation (cellular component strategies), nucleoplasm part, non-membrane-bounded organelle, and chromosome organization (cellular component categories), and nucleoside phosphate binding (molecular function categories). Furthermore, we also performed KEGG analysis (Fig. 5b, Supplementary excel file 4 and Table S7), Hippo signaling pathway, oxidative phosphorylation, metabolic pathways, and glycerolipid metabolism were up-regulated, and lysine degradation, sucrose, and starch metabolism and neuroactive ligand-receptor interaction were down-regulated.

**Figure 6:**
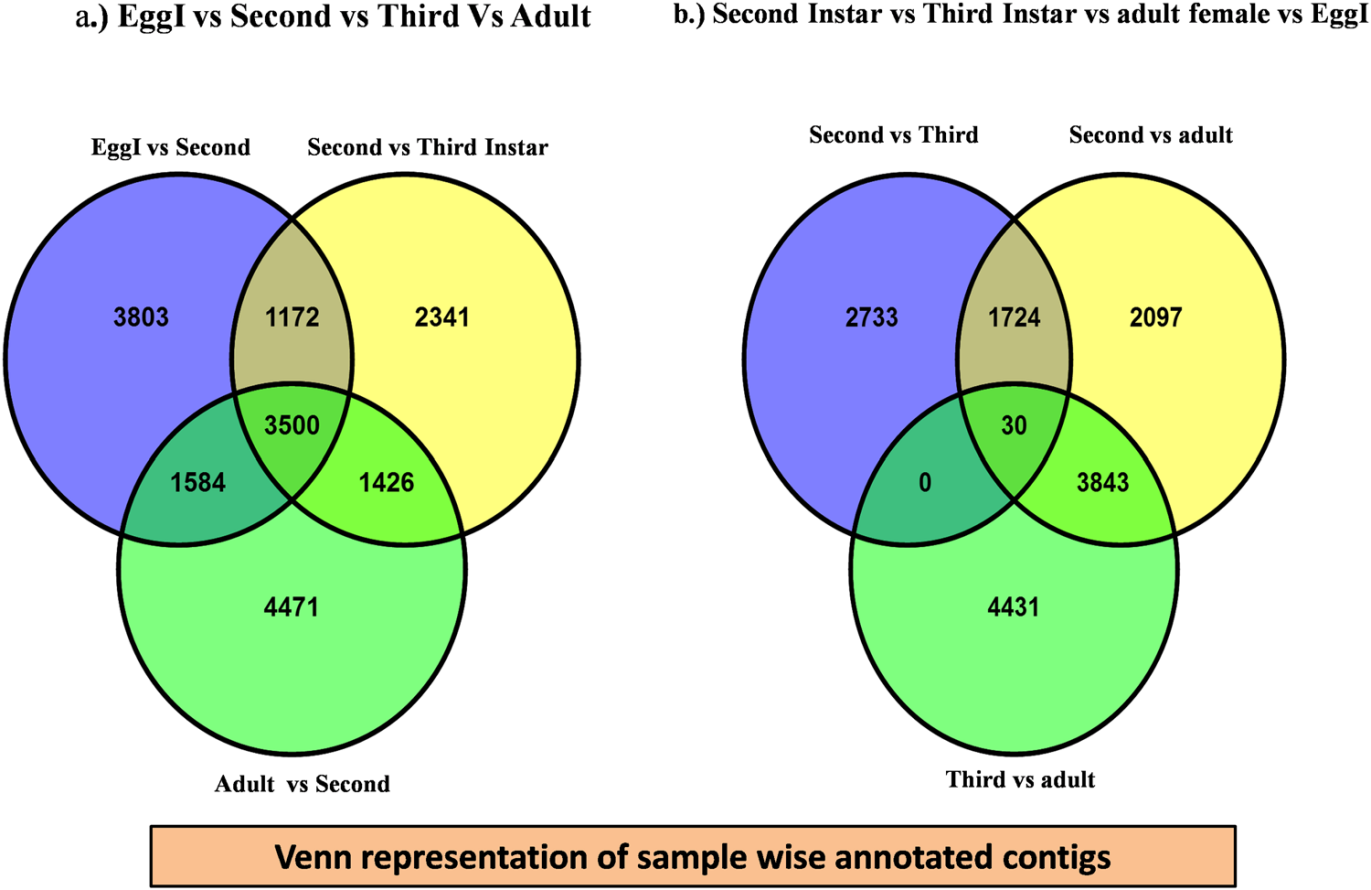
Venn diagrams representing number of differentially expressed genes among developmental stages. (**a**) Number of genes differentially expressed between the EggI vs. Second Instar vs. Third Instar vs. Adult female, commonly shared or not among stages. (**b**) Number of genes expressed between Second vs. Third vs. adult female, commonly shared or not among stages.

Venn diagrams of the DEG’s among developmental stages allowed the identification of transcripts exclusive or common to the various stages of *P. solenopsis*. The first Venn diagram comprising DEG’s between EggI and all analyzed instar identified 10,059, 8,439 and 10,981 DEG’S between EggI and the second, third and adult stages, respectively. By comparing, the EggI with second instar, third instar, and adult 3,500 DEG’s were identified that shared between all EggI, second, third and adult female stages (Fig. 6a). Of the 10,059 DEG’S between EggI and second instar, 3803 were exclusive whereas 1172 DEG’s were shared between second and third instar. The remaining was shared among the second, third and adult female (Fig. 6a). Of the 8439 DEG’s between EggI and 3rd instar, 2341 were exclusive, 1426 were shared between the third and adult stages, and 1584 were shared between the second and adult female stages (Fig. 6a).

A Venn diagram of DEG’S between second, third instar stages showed that there were 4,487, 7694 and 8304 DEG’s between second and third, adult female stages, respectively (Fig. 6b). In this case, 30 DEG’S were commonly found between second, third and adult stages (Fig. 6b). Of the 4487 DEG’S between second and the third instar stage, 2733 were exclusive (Fig. 6b). Of the 7694 DEG’S between adults and second stage, 2097 were exclusive, and 3843 were also differentially expressed between adults and third instar. Of the 4487 DEG’S between second and third instar stage, 4431 were exclusive (Fig. 6b). Among the 587 DEG’S between adults and EggI stage, 117 were exclusive to this stage (Fig. 6b).

The euclidian distance matrix was estimated using normalized gene expression values of all the libraries based on transcripts profiles to create a dendrogram and a heatmap describing the similarities among the developmental stages of *P. solenopsis* (Supplementary Fig. S2). The heatmap and the clustered groups clearly indicated a similarity gradient between the developmental stages, with the transcript profile from second instar more similar to that of the third instar, and from the third instar more similar to the adult females. The gene expression profile from EggI was the most distinct from all the other stages, particularly from adults. There was less similarity among EggI and second instar than for third instar and an adult female. Among the different instars, the closer the stages, the higher the similarity of transcripts profiles.

### Validation of differently expressed transcripts (DEG’S) during development using qRT-PCR

**Figure 7:**
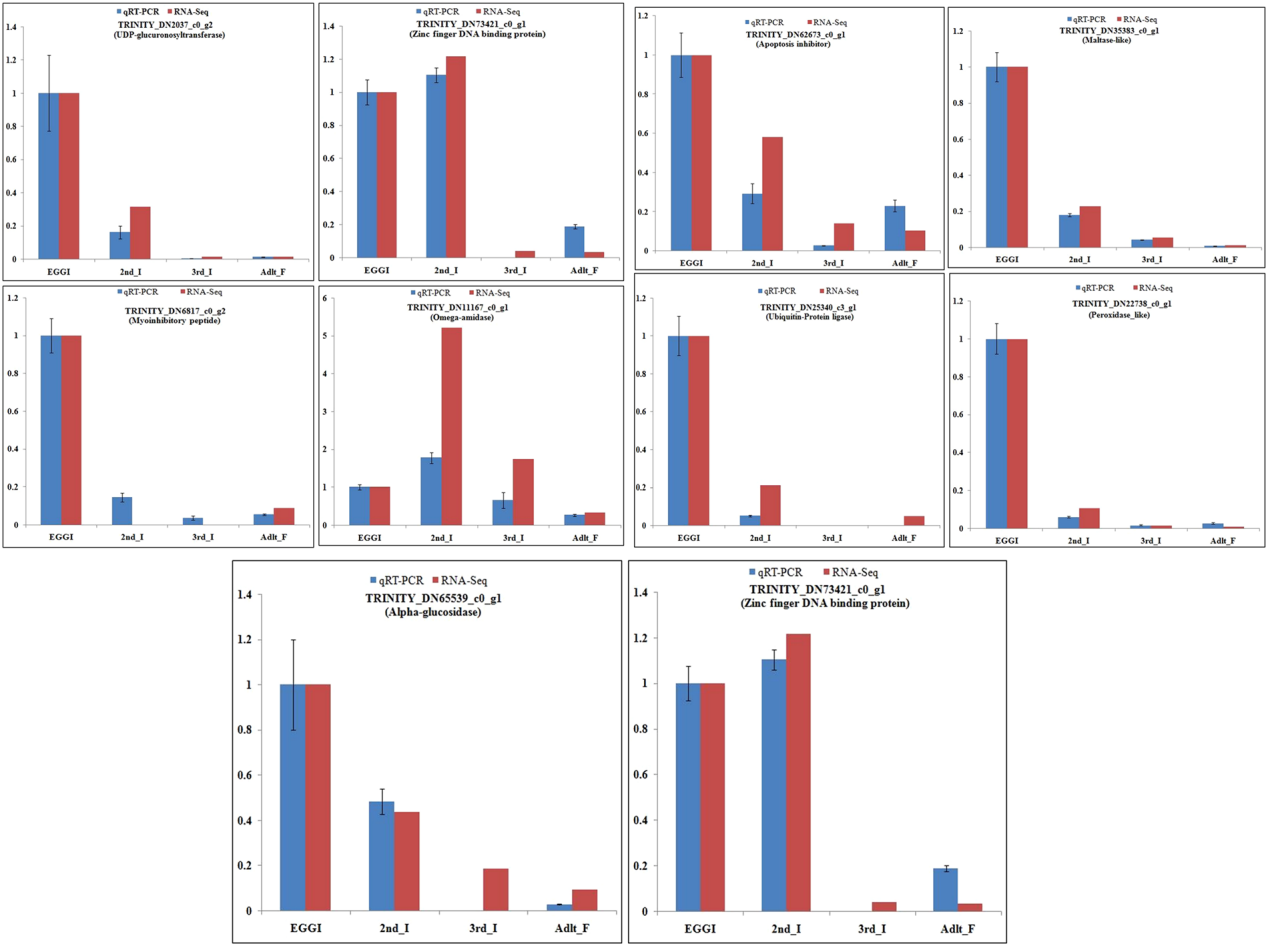
Comparison of qRT-PCR (red bar) and transcriptome (blue bar) expression data for ten randomly selected genes to confirm expression patterns indicated by transcriptome sequencing. Three technical replicates were performed for each of the three biological replicates. The height of each bar chart represents the mean average of sample-specific 2^−ΔΔCt^ values. “1” represents the egg stage, “2” represents the 2nd Instar stage, “3” represents the 3rd instar stage, and “4” represents the adult stages.

qRT-PCR analyses were performed on ten randomly selected genes: TRINITY_DN6817_c0_g2, TRINITY_DN5360_c0_g1, TRINITY_DN25340_c3_g1, TRINITY_DN22738_c0_g1, TRINITY_DN11167_c0_g1, TRINITY_DN62673_c0_g1, TRINITY_DN35383_c0_g1, TRINITY_DN2037_c0_g2, TRINITY_DN65539_c0_g1 and TRINITY_DN73421_c0_g1. (Fig. 7 and Table S1). TRINITY_DN6817_c0_g2, TRINITY_DN5360_c0_g1, TRINITY_DN25340_c3_g1, TRINITY_DN22738_c0_g1, TRINITY_DN11167_c0_g1, TRINITY_DN62673_c0_g1, TRINITY_DN35383_c0_g1, and TRINITY_DN65539_c0_g1 were primarily expressed in all stages, whereas TRINITY_DN2037_c0_g2 & TRINITY_DN73421_c0_g1 were not showing any expression in adult stage. The high confirmation rate of the unigenes indicated the reliability of the transcriptome data.

### Identification of differentially expressed genes involved in hormone biosynthesis

**Figure 8:**
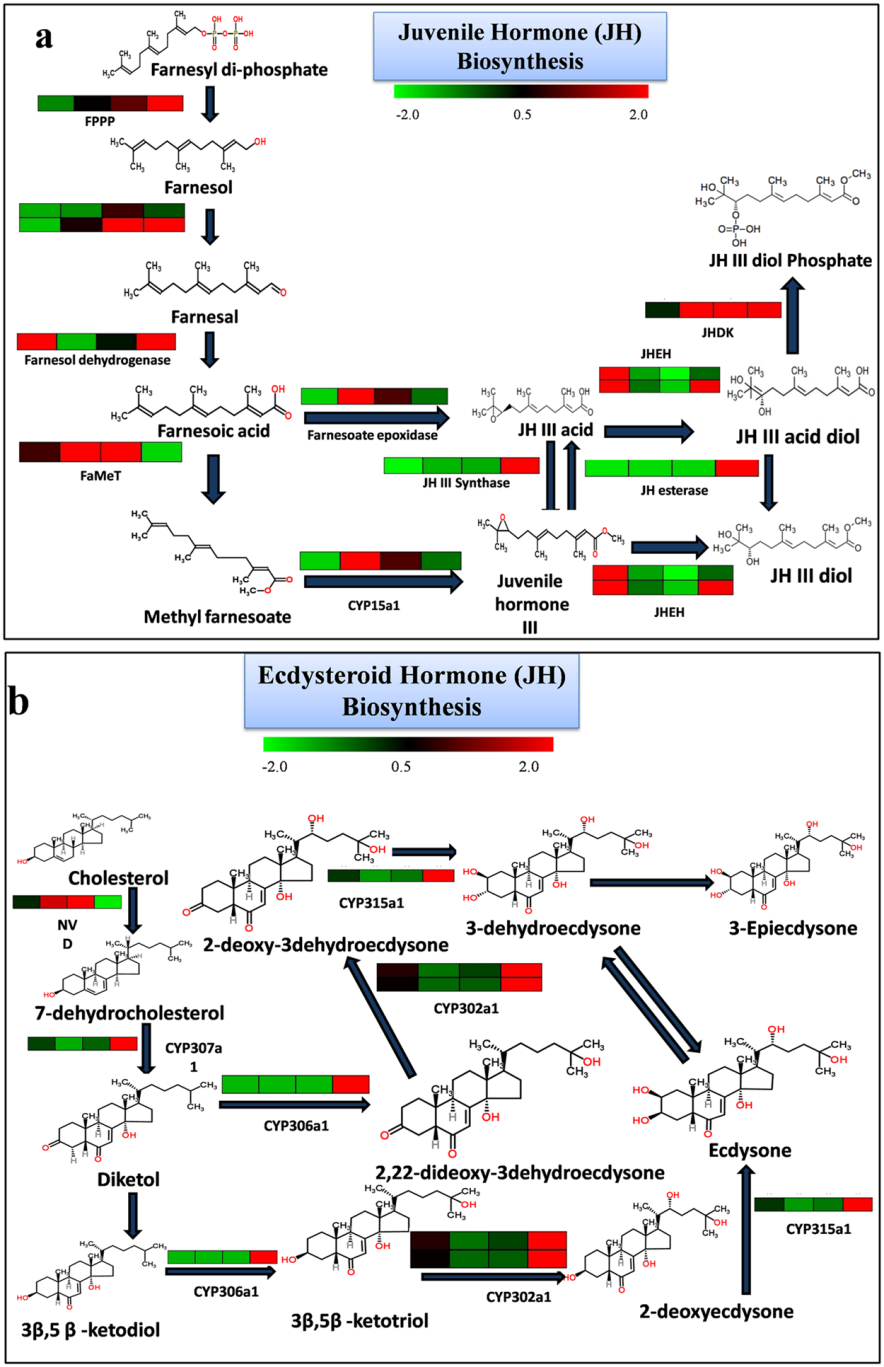
Juvenile hormone and ecdysteroid hormone biosynthesis with number of hits from the *P. solenopsis* transcriptome. (**a**) The analysis of the transcriptome identified homologues genes coding for enzymes of the Juvenile Hormone biosynthetic pathway, including Farnesylpyrophosphate synthase (Fpps); Juvenile Hormone Acid Methyltransferase (Jhamt); and Cytochrome P450 (CYP15A1); and for degradation of the Juvenile Hormone, with Juvenile Hormone Epoxide Hydrolase (Jheh); Juvenile Hormone Esterase 1 (jhe1), with respective number of identified hits at e < e−30 by Blastx. Chemical structures obtained from KEGG. (**b**) The analysis of the transcriptome identified homologues genes coding for enzymes of the Ecdysteoid hormone biosynthetic pathway, including Cholesterol 7-dehydrogenase (Neverland); and a series of cytochrome P450 enzymes encoded by the Halloween genes, such as spook (CYP307A); phantom (CYP306A1); disembodied (CYP302A1); shadow (CYP315A1); and shade (CYP314A1). For ecdysteroid inactivation, a 26-dehydroxylase (CYP18A1), with respective number of identified hits at e < e−30 by Blastx. Chemical structures obtained from KEGG.

The contigs related to the genes involved in the biosynthesis of sesquiterpenoid juvenile hormone (JH) and ecdysteroid pathways were identified by blast search against the reported genes from *A. pisum*. For JH II biosynthesis, six contigs were mapped to farnesylpyrophosphate synthase genes (Fps, Fpps2), four to juvenile hormone acid methyltransferase (Jhamt), and twelve to cytochrome P450 family 15, subfamily A, polypeptide 1 (CYP15A1) of *A. pisum* with significant similarity (identity > 60% and E < e−10). For JH III degradation, thirteen contigs showed similarity to juvenile hormone esterase (Jhe), thirteen to juvenile hormone epoxide hydrolase (Jheh), and twenty-one to juvenile hormones esterase-like protein (Jdhk) (Fig. 8a). List of identified contigs and their expression values is shown in Table S8.

In case of ecdysteroid biosynthesis, two contigs were identified for neverland gene (cholesterol dehydrogenase). A subsequent search was performed to identify the contigs for cytochrome P450 enzymes encoded by halloween genes. A total of thirteen contigs showed similarity to spook (CYP307A), eleven to phantom (CYP306A1), nine to disembodied (CYP302A1), nine to shadow (CYP315A1), and eleven to shade (CYP314A1). For ecdysteroid inactivation, fourteen contigs showed similarity to 26-dehydroxylase (CYP18A1), which is putatively responsible for 20, 26-dihydroxyecdysone production. Twenty contigs showed similarity with 3-dehydroecdysone-3-α-reductase and fifteen with ecdysone oxidase, which is putatively responsible for 3-epiecdysone production, were identified (Fig. 8b). Complete information about the gene listed for ecdysteroid biosynthesis with mapped contigs and their expression values are given in Table S8.

## Discussion

The mealybug, *P. solenopsis* is a major invasive pest of cotton destroying a vast range of agriculturally important cash crop. However, their detail genomics study has not been done so far. In the absence of complete genome sequences, de novo-transcriptome analysis can help in estimating future gene expression and functional analysis on *P. solenopsis*. That can also enhance our understanding of its biological processes and molecular mechanisms. In the current study, RNA-Seq technology was applied to reveal the comparative transcriptome profiling of four developmental stages (EggI, second, third and adult female) of *P. solenopsis*. The de-novo assembly of short read sequence without having reference genome poses a challenge with existing bioinformatics tools^39^. Here, we used trinity software which is a well-accepted methodology for its precision and quality of the assembled sequences^29^. Before doing assembly, the four datasets (EggI, second, third instar and adult female) were merged, and read abundance estimation was normalized to 50 × coverage using the *in-silico* normalization tool of Trinity to improve assembly duration and minimize the memory need. Filtering and normalization reduced the dataset to 43.59 GB. About 182.67 million normalized read pairs were assembled using Trinity at default parameters. The 38,725 contigs annotated using NR database showed the highest similarity of *P. solenopsis* with the *A. pisum* rather than *B. tabaci*. This disparity was observed due to the close evolutionary relationship between *A. pisum* and *P. solenopsis*, which referred a common selection of genes between closely related insects. This pattern is different from the transcriptome studies of hemipterans, *B. tabaci*^40,41^, *A. mellifera*^42^, *Myzus persicae*^43^ and *A. gossypii*^44^. These observations are likely due to non-availability of sufficient transcriptome data for the hemipteran species published during the study period.

Further, we compared the gene expression profiles between four developmental stages by constructing DEGs libraries, which revealed 29,415 differentially expressed genes. The total number of unigenes of the *de-novo* assembled assembly was higher than expected total number of genes found in other hemiptera and lepidoptera species^41,43,45^. Recently, an article was published that showed transcriptome data analysis of *P. solenopsis* to identify SSR markers and genes involved in sex pheromone metabolism^46,47^. The reported study was basically done on female adult mealybugs, but no comparative differential analysis was performed on the insect developmental stages and hormone biosynthesis. The present study is the first report on *P. solenopsis* that explained the differential expression of the genes involved in insect development and hormone biosynthesis pathway. The information generated through the analysis provides molecular resources for the study of other hemipteran and related species that establish a framework to interpret the changes in gene expression during insect development.

An interesting finding from this study is the identification of many differentially expressed genes across the developmental stages that enriched current knowledge of *P. solenopsis* gene expression profiles. It will be useful for future research related to various developmental, physiological and metabolic pathways in this as well as in other related insects. It may also help to identify vital RNAi target for the control of *P. solenopsis*, as reported in the case of other hemipteran insects^48–51^. During comparison between different developmental libraries, a large number of genes showed specific life stage-related expression profiles (e.g., ecdysteroid and juvenile hormone related) that were likely involved in developmental differentiations. The developmental characteristics of *P. solenopsis* are very typical and showed hemi-metabolous metamorphosis; which means insects develop from eggs to first instar nymphs, and then to second-third instar, eventually emerge as either adult male or female^4,5,52^. The Adult female is considered as the most devastating stage for infesting host plants that lay eggs and start a new life cycle^4,53^. During the developmental growth from eggs to first–second instars, 10,059 up-regulated genes and 21,581 down-regulated genes, third to adult stage showed 4,487 up-regulated genes, and 24,224 down-regulated genes were found. From the available differential expressed genes (DEGs), the search was performed to identify the ten-most up-regulated and down-regulated genes in different developmental comparison. The top up-regulated genes in EggI to adult female stage was found to be putative antimicrobial knottin protein of *B. tabaci*^54^, eukaryotic translation initiation factor 3, serine proteinase of *P. humanus corporis*^55^, *DnaJ domain-containing protein of B. mori*^56–58^, 60 S ribosomal protein L18 of *A. pisum*, tubulin beta-3 chain-like of *A. pisum*^59^, Rad21 CG17436-PA of *T. castaneum*^60,61^, sodium-independent sulfate anion transporter-like isoform X1 of *Apis dorsata*^62^ and putative antimicrobial knottin protein Btk-4 of *B. tabaci*. The down-regulated genes were tumor protein p63 isoform alpha 2-like genes of *A. pisum*, similar to Secretory Phospholipase A2^63,64^ of *T. castaneum*, zinc finger protein SLUG^65^ of *P. humanus corporis*, regulator of G-protein signaling 9-like isoform 1^66,67^ of *P. humanus corporis*, and chromobox protein homolog 5-like isoform 1^68^ of *Nasonia vitripennis*. Furthermore, to identify significant GO categories and KEGG pathways in different developmental stages of *P. solenopsis* GO, and KEGG enrichment analyses were performed. GO enrichment analysis show that up regulated developmental specific genes belong to ‘protein metabolic process’, ‘biosynthetic process’, ‘organic substance metabolic process’, ‘ribosome biogenesis’, ‘protein binding’, ‘structural molecule activity’ and ‘protein targeting’. According to the KEGG pathway analysis, fifteen, six, and eleven pathways were observed as significantly enriched for DEGs of EggI vs. second instar, second vs. third instar, and third instar vs. adult females, respectively. The pathway enriched for different developmental stages were glycerolipid metabolism^69^, FoxO-signaling pathway^20,70–73^, Hippo signaling pathway^74–76^, fatty acid biosynthesis^77–79^, biosynthesis of amino acids^41,80–85^ and steroid biosynthesis^86–88^ suggesting that genes in these pathways seem to be involved in early developmental process. Likewise, we have identified all the genes coding for enzymes involved in juvenile and ecdysteroid hormone biosynthetic pathways and found many of them as potential RNAi targets for silencing (Fig. 8). In JH III biosynthesis pathway, there were twelve to twenty contigs homologous to each gene from *A. pisum* and *M. hirsutus*, while 10–13 contigs were recognized for the degrading enzymes for JHE and JHEH. In case of ecdysteroid biosynthesis which completely depends on sterols obtained from the diet, involves consecutive action of dehydrogenase (neverland), followed by a series of cytochrome P450 enzymes encoded by the halloween genes. In the insect hormone biosynthesis pathway, all the genes that encode enzymes of the ecdysteroid and sesquiterpenoid juvenile hormone biosynthetic pathways can be a vital target for RNAi and other silencing mechanism. By regulating the developmental pathway gene expression would be a challenging and attractive RNAi method to control the invasiveness of sap-sucking pest, *P. solenopsis*.

This study generated four transcriptome libraries and gave information regarding the genomic resources for further evaluation of *P. solenopsis*. The information generated through analyses will help in future to identify the potential targets in relation to developmental pathways. Moreover, many unigenes and expression profiles of high-quality transcriptome datasets of *P. solenopsis* serve as a step towards understanding the role of different stages in the spread of mealybug and their infestation on agriculturally important cash crops for their control.

## Electronic supplementary material

Supplementary excel file 1

Supplementary excel file 2

Supplementary excel file 3

Supplementary excel file 4

Supplementary file
