# Supplementary material for: De novo characterization of Phenacoccus solenopsis transcriptome and analysis of gene expression profiling during development and hormone biosynthesis
Source: Sci Rep. 2018 May 15;8:7573. doi: 10.1038/s41598-018-25845-3 (PMC5954142; doi:10.1038/s41598-018-25845-3)
Supplement: Supplementary file 5 — Supplementary file [file 41598_2018_25845_MOESM5_ESM.docx]

***De novo* characterization of *Phenacoccus solenopsis* transcriptome and analysis of gene expression profiling during development and hormone biosynthesis**

Surjeet Kumar Arya^1,2^, Yogeshwar Vikram Dhar^1,2^, Santosh Kumar Upadhyay^3^, Mehar Hasan Asif^1,2^, Praveen Chandra Verma^1, 2^*

^1^CSIR-National Botanical Research Institute, (Council of Scientific and Industrial Research) Rana Pratap Marg, Lucknow, UP-226001, India.

^2^Academy of Scientific and Innovative Research (AcSIR), Anusandhan Bhawan, Room No: 310, 2-Rafi Marg, New Delhi, India.

^3^Department of Botany, Panjab University, Chandigarh, India-160014.

***Corresponding Author**

**Praveen Chandra Verma**

CSIR-National Botanical Research Institute,

Council of Scientific and Industrial Research

Rana Pratap Marg, Lucknow, UP, India.

Tel: +91-0522-2297922

Fax: +91-0522-2205836, 2205839

Email: praveencverma@nbri.res.in

**Figure S1.** *De-novo* transcriptome transcriptome assembly workflow.


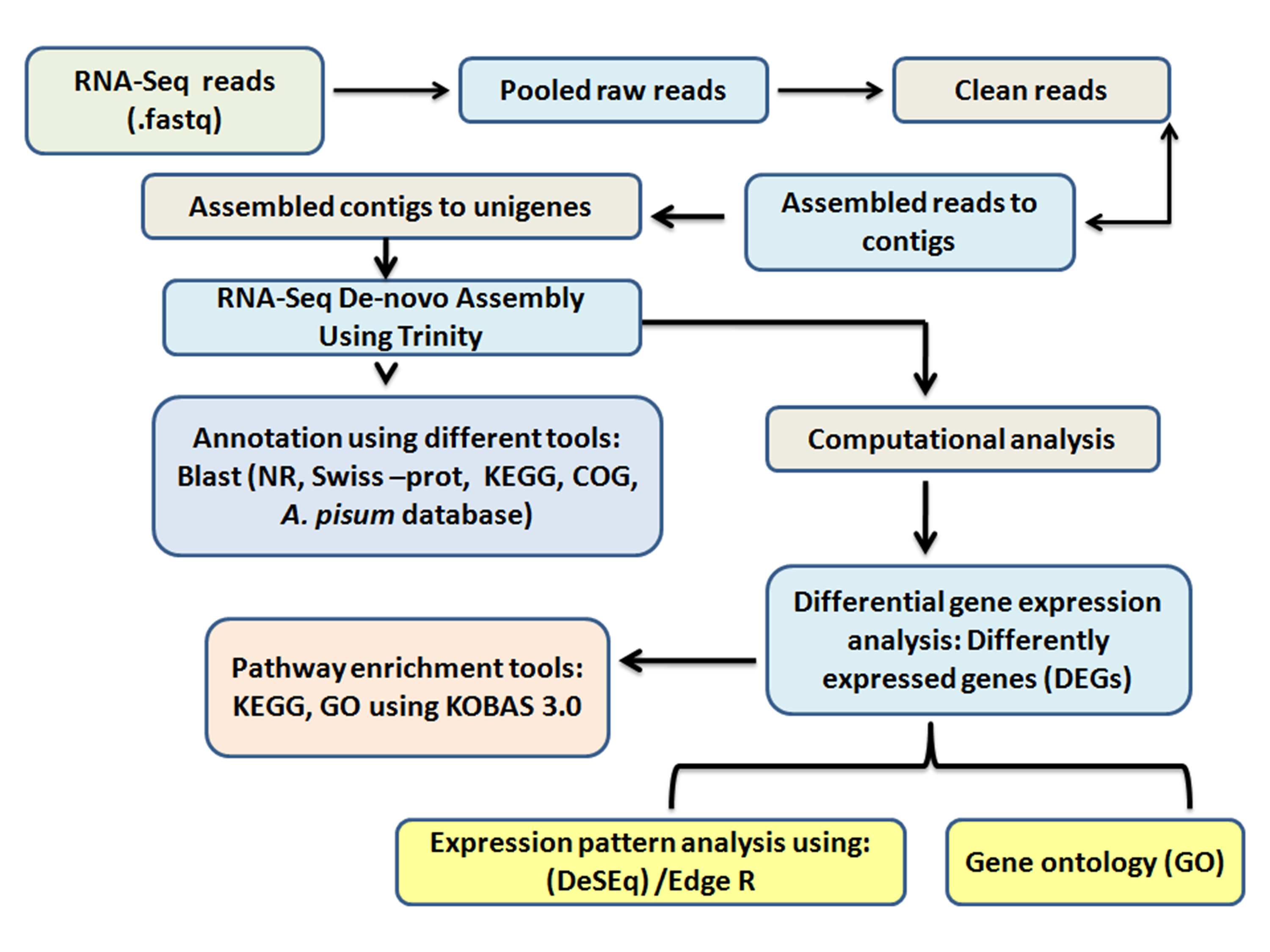


**Figure S2.** Heat map and clustering representing most similar transcriptome profiles among *P. solenopsis* developmental stages. The count matrix for all sequenced samples was used to calculate an Euclidian distance matrix, which was used for hierarchical sample clustering, according to the most similar transcriptome profile using the single linkage method, to generate a dendrogram and a heatmap correlating all sample expression profiles into colors, ranging from red (identical profiles) to green (most different). The developmental stages analyzed were egg (EggI); 2nd stage instar (Second_Instar); 3rd stage instar (Third_Instar); and adult female (AD).


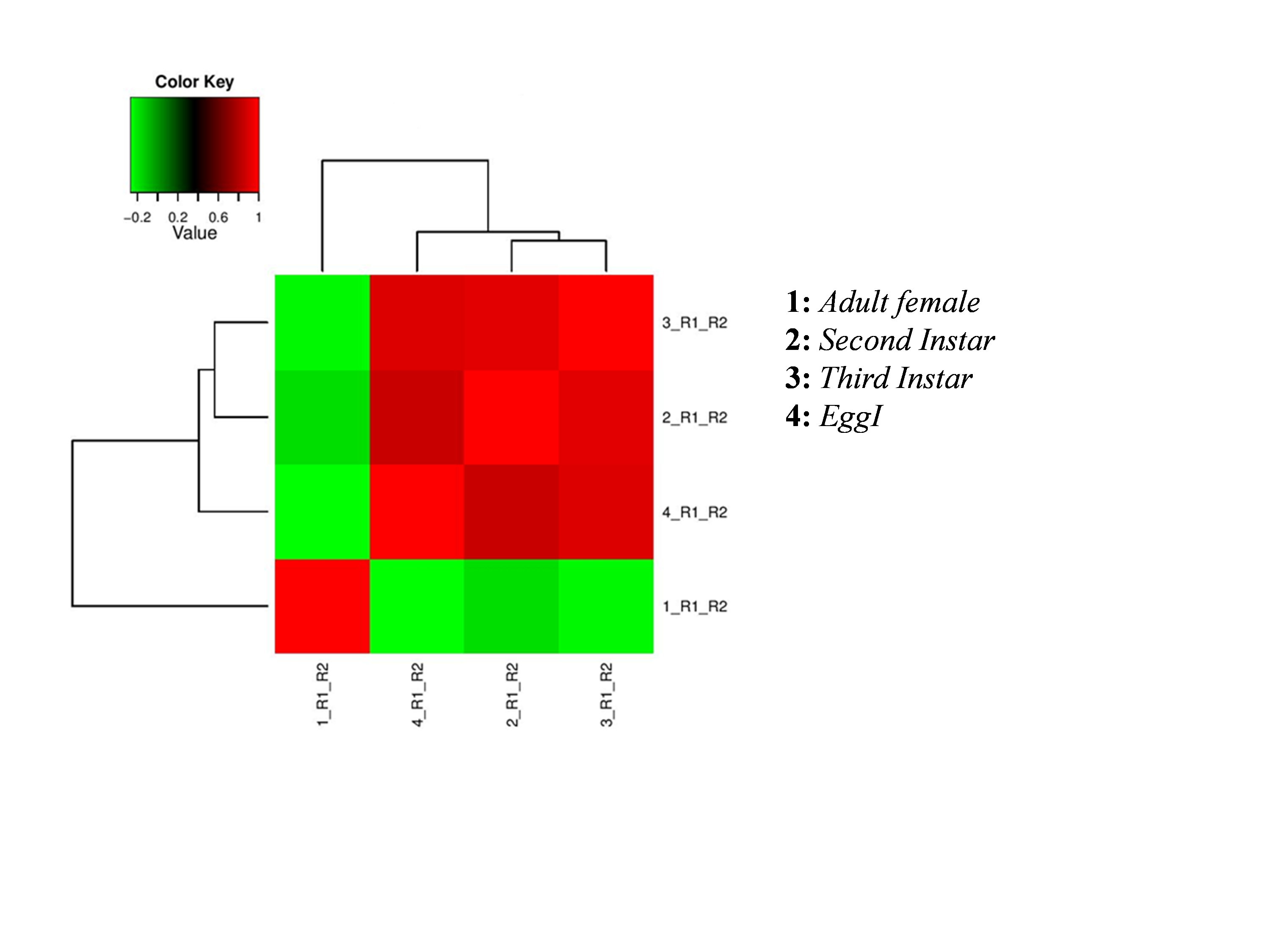


**Table S1** Primer sequences of 10 genes differentially expressed between the developmental stages with expected product size, and three gene references used in qRT-PCR to validate differences in read counts among libraries.

| **Gene_ID** | **Cellular Function** | **Primers** | **Amplicon length** |
| --- | --- | --- | --- |
| TRINITY_DN11167_c0_g1 | omega-amidase NIT2 | F’-5-GGAACGTCCACGTTGAATAAGTGT-3  F’-5-GTAGTCGCTGGAACCATCCC-3’ | 125 |
| TRINITY_DN62673_c0_g1 | probable ATP-dependent RNA helicase | F’-5-CAGACGTGGTTTCGGACTATGT-3’  R’-5- GGTATTTGAGGGTAAGAACTTCGTGA-3’ | 80 |
| TRINITY_DN6817_c0_g2 | myoinhibitory peptide | F’-5-CAAATGTTGACGAGTGCCG-3’  R’-5- TTCGCCTCGTGTGTTATTGC-3’ | 111 |
| TRINITY_DN5360_c0_g1 | fatty acid synthase-like | F’-5-ACCACGTCACCCATACAGAGGATAC-3’  F’-5-GCCATTGAGAACCCATTCCG-3’ | 131 |
| TRINITY_DN35383_c0_g1 | probable maltase L-like | F’-5-TGCATCAGTTCTCCGTGAGTCAAC-3’  F’-5-ATAAAATGTGTCGGAGCGTCCAG-3 | 136 |
| TRINITY_DN2037_c0_g2 | UDP-glucuronosyltransferase | F’-5-CACGTTTTTCGGCATGTCAT-3’  F’-5-CCTTCGTCACTGGCACCC-3’ | 114 |
| TRINITY_DN25340_c3_g1 | E3 ubiquitin-protein ligase Nedd-4-like | F’-5-GTTTCCAATAGCAAGAATCCCAA-3’  F’-5-ACAGACACCCGAAGACAAGAAAC-3’ | 157 |
| TRINITY_DN22738_c0_g1 | peroxidase-like | F’-5-TCGTGTGCTAGGAGCTTCGC-3’  F’-5-GGGATGCCAAACAACACCGT-3’ | 168 |
| TRINITY_DN65539_c0_g1 | alpha-glucosidase-like | F’-5-AACCACGGGTGTTCAATGCT-3’  F’-5-TGTCGGATTTCATTTCTATAGCTCC-3’ | 139 |
| TRINITY_DN73421_c0_g1 | zinc finger DNA binding protein | F’-5-GCGTCCCTTATCGCAATGA-3’  F’-5-GGTATCTTTCCCTCTGAGCTAGGTA-3’ | 130 |

**Table S2** Species distribution statistics in *Phenacoccus solenopsis* transcriptomes.

| *Acyrthosiphon pisum* | 4182 |
| --- | --- |
| *Tribolium castaneum* | 1933 |
| *Pediculus humanus corporis* | 1126 |
| *Nasonia vitripennis* | 621 |
| *Aspergillus oryzae RIB40* | 425 |
| *Megachile rotundata* | 420 |
| *Bombyx mori* | 381 |
| *Camponotus floridanus* | 356 |
| *Maconellicoccus hirsutus* | 343 |
| *Apis mellifera* | 288 |
| *Harpegnathos saltator* | 271 |
| *Dendroctonus ponderosae* | 256 |
| *Bombus terrestris* | 253 |
| *Aedes aegypti* | 216 |
| *Acromyrmex echinatior* | 210 |
| *Bombus impatiens* | 209 |
| *Aspergillus flavus NRRL3357* | 204 |
| *Riptortus pedestris* | 202 |
| *Other* | 7226 |
| *Solenopsis invicta* | 198 |
| *Danaus plexippus* | 182 |
| *Apis dorsata* | 175 |
| *Rickettsiaceae bacterium Os18* | 161 |
| *Apis florea* | 159 |
| *Nectria haematococca mpVI 77-13-4* | 158 |
| *Culex quinquefasciatus* | 137 |
| *Musca domestica* | 137 |
| *Anopheles gambiae str. PEST* | 131 |
| *Rickettsia endosymbiont of Ixodes scapularis* | 118 |
| *Daphnia pulex* | 117 |
| *Saccoglossus kowalevskii* | 110 |
| *Ceratitis capitata* | 109 |
| *Branchiostoma floridae* | 103 |
| *Capitella teleta* | 95 |
| *Hydra vulgaris* | 91 |
| *Mus musculus* | 90 |
| *Drosophila melanogaster* | 89 |
| *Lottia gigantea* | 85 |
| *Fusarium graminearum PH-1* | 81 |
| *Fusarium oxysporum Fo5176* | 74 |
| *Fusarium fujikuroi IMI 58289* | 73 |
| *Anopheles darlingi* | 72 |
| *Nilaparvata lugens* | 71 |
| *Biomphalaria glabrata* | 68 |
| *Brugia malayi* | 68 |
| *Ixodes scapularis* | 64 |
| *Strongylocentrotus purpuratus* | 62 |
| *Metaseiulus occidentalis* | 59 |
| *Drosophila ananassae* | 56 |
| *Coptotermes formosanus* | 55 |
| *Aspergillus oryzae 3.042* | 53 |
| *Aplysia californica* | 50 |
| *Danio rerio* | 48 |
| *Locusta migratoria* | 47 |
| *Amphimedon queenslandica* | 45 |
| *Drosophila virilis* | 45 |
| *Fusarium verticillioides 7600* | 44 |
| *Vitis vinifera* | 44 |
| *Drosophila pseudoobscura pseudoobscura* | 43 |
| *Crassostrea gigas* | 41 |
| *Xenopus (Silurana) tropicalis* | 41 |
| *Dothistroma septosporum NZE10* | 40 |
| *Helobdella robusta* | 38 |
| *Baudoinia compniacensis UAMH 10762* | 36 |
| *Fusarium pseudograminearum CS3096* | 35 |
| *Drosophila willistoni* | 33 |
| *Aphis gossypii* | 32 |
| *Arabidopsis thaliana* | 32 |
| *Drosophila mojavensis* | 32 |
| *Nematostella vectensis* | 32 |
| *Clonorchis sinensis* | 31 |
| *Sus scrofa* | 31 |
| *Medicago truncatula* | 29 |
| *Bos mutus* | 28 |
| *Oreochromis niloticus* | 28 |
| *Portunus trituberculatus* | 27 |
| *Drosophila grimshawi* | 26 |
| *Haemonchus contortus* | 26 |
| *Homo sapiens* | 26 |
| *Schistosoma mansoni* | 26 |
| *Oryza sativa Japonica Group* | 25 |
| *Zymoseptoria tritici IPO323* | 25 |
| *Glycine max* | 24 |
| *Gryllus bimaculatus* | 24 |
| *Latimeria chalumnae* | 24 |
| *Trichinella spiralis* | 24 |
| *Drosophila yakuba* | 23 |
| *Fusarium oxysporum f. sp. cubense race 4* | 23 |
| *Rattus norvegicus* | 23 |
| *Planococcus citri* | 22 |
| *Pseudocercospora fijiensis CIRAD86* | 22 |
| *Trichomonas vaginalis G3* | 22 |
| *Ciona intestinalis* | 21 |
| *Oryzias latipes* | 21 |
| *Periplaneta americana* | 21 |
| *Trichoplax adhaerens* | 21 |
| *acceptor* | 0 |
| *Anolis carolinensis* | 20 |
| *Caenorhabditis remanei* | 20 |
| *Drosophila persimilis* | 20 |
| *Tremella mesenterica DSM 1558* | 20 |
| *Drosophila simulans* | 19 |
| *Lepisosteus oculatus* | 19 |
| *Aphis citricidus* | 18 |
| *Caenorhabditis brenneri* | 18 |
| *Hymenolepis microstoma* | 18 |
| *Bos taurus* | 16 |
| *Fusarium oxysporum FOSC 3-a* | 16 |
| *Papilio xuthus* | 16 |
| *Chelonia mydas* | 15 |
| *Escherichia coli* | 15 |
| *Simkania negevensis Z* | 15 |
| *Aphis glycines* | 14 |
| *Cricetulus griseus* | 14 |
| *Drosophila erecta* | 14 |
| *Drosophila sechellia* | 14 |
| *Hordeum vulgare subsp. vulgare* | 14 |
| *Reticulitermes flavipes* | 14 |
| *Rickettsia helvetica* | 14 |
| *Bemisia tabaci* | 13 |
| *Cryptococcus gattii WM276* | 13 |
| *Helicoverpa armigera* | 13 |
| *Macaca fascicularis* | 13 |
| *ubiquinone* | 13 |
| *Zea mays* | 13 |
| *Aspergillus terreus NIH2624* | 12 |
| *Chrysemys picta bellii* | 12 |
| *Gallus gallus* | 12 |
| *Sphaerulina musiva SO2202* | 12 |
| *Anopheles gambiae* | 11 |
| *Blattella germanica* | 11 |
| *Equus caballus* | 11 |
| *Ericerus pela* | 11 |
| *Laodelphax striatella* | 11 |
| *Populus trichocarpa* | 11 |
| *Takifugu rubripes* | 11 |
| *Xiphophorus maculatus* | 11 |
| *Aspergillus oryzae* | 10 |
| *Coniosporium apollinis CBS 100218* | 10 |
| *Falco cherrug* | 10 |
| *Fusarium oxysporum f. sp. cubense race 1* | 10 |
| *Macrophomina phaseolina MS6* | 10 |
| *Mesembryanthemum crystallinum* | 10 |
| *Myotis brandtii* | 10 |
| *Necator americanus* | 10 |
| *Phaseolus vulgaris* | 10 |
| *Caenorhabditis briggsae* | 9 |
| *Candidatus Rickettsia amblyommii str. GAT-30V* | 9 |
| *Cowpea mild mottle virus* | 9 |
| *Gluconacetobacter sp. SXCC-1* | 9 |
| *Neolamprologus brichardi* | 9 |
| *Orientia tsutsugamushi str. Ikeda* | 9 |
| *Pantholops hodgsonii* | 9 |
| *Schistocerca gregaria* | 9 |
| *Schistosoma japonicum* | 9 |
| *Theobroma cacao* | 9 |
| *Trichosporon asahii var. asahii CBS 2479* | 9 |
| *Xenopus laevis* | 9 |
| *Cicer arietinum* | 8 |
| *Citrus clementina* | 8 |
| *Citrus sinensis* | 8 |
| *Guillardia theta CCMP2712* | 8 |
| *Heterocephalus glaber* | 8 |
| *Nomascus leucogenys* | 8 |
| *Ochotona princeps* | 8 |
| *Rhodnius prolixus* | 8 |
| *Sarcophilus harrisii* | 8 |
| *Tetrahymena thermophila* | 8 |
| *Tupaia chinensis* | 8 |
| *Vicia faba* | 8 |
| *Alligator sinensis* | 7 |
| *Anas platyrhynchos* | 7 |
| *Aspergillus fumigatus Af293* | 7 |
| *Bactericera cockerelli* | 7 |
| *Callorhinchus milii* | 7 |
| *Cotesia congregata bracovirus* | 7 |
| *Dasypus novemcinctus* | 7 |
| *Elephantulus edwardii* | 7 |
| *Fusobacterium nucleatum* | 7 |
| *Lotus japonicus* | 7 |
| *Mesocricetus auratus* | 7 |
| *Metarhizium anisopliae ARSEF 23* | 7 |
| *Microtus ochrogaster* | 7 |
| *Morus notabilis* | 7 |
| *Mustela putorius furo* | 7 |
| *Myzus persicae* | 7 |
| *Neofusicoccum parvum UCRNP2* | 7 |
| *Papilio polytes* | 7 |
| *Pteropus alecto* | 7 |
| *Pundamilia nyererei* | 7 |
| *Pyrenophora tritici-repentis Pt-1C-BFP* | 7 |
| *Sesuvium portulacastrum* | 7 |
| *synthetic construct* | 7 |
| *Aedes albopictus* | 6 |
| *Ailuropoda melanoleuca* | 6 |
| *Aster yellows phytoplasma* | 6 |
| *Chrysochloris asiatica* | 6 |
| *Clostridium lentocellum DSM 5427* | 6 |
| *Columba livia* | 6 |
| *Condylura cristata* | 6 |
| *Cryptococcus neoformans var. neoformans JEC21* | 6 |
| *Enterobacteria phage lambda* | 6 |
| *Fusarium oxysporum f. sp. pisi HDV247* | 6 |
| *Genlisea aurea* | 6 |
| *Glyptapanteles flavicoxis* | 6 |
| *Gossypium hirsutum* | 6 |
| *Haplochromis burtoni* | 6 |
| *Liposcelis bostrychophila* | 6 |
| *Maylandia zebra* | 6 |
| *Orientia tsutsugamushi str. Boryong* | 6 |
| *Oxytricha trifallax* | 6 |
| *Pelodiscus sinensis* | 6 |
| *Rhizopus delemar RA 99-880* | 6 |
| *Schistocerca americana* | 6 |
| *Triatoma infestans* | 6 |
| *Antheraea pernyi* | 5 |
| *Aspergillus clavatus NRRL 1* | 5 |
| *Aspergillus nidulans FGSC A4* | 5 |
| *Beta vulgaris* | 5 |
| *Beta vulgaris subsp. maritima* | 5 |
| *Camelus ferus* | 5 |
| *Cladosporium herbarum* | 5 |
| *Cryptococcus neoformans var. grubii H99* | 5 |
| *Curvibacter putative symbiont of Hydra magnipapillata* | 5 |
| *Cu-Zn* | 5 |
| *Diaphorina citri* | 5 |
| *Eutrema salsugineum* | 5 |
| *Fenneropenaeus chinensis* | 5 |
| *Ficedula albicollis* | 5 |
| *Fusarium fujikuroi* | 5 |
| *Fusobacterium periodonticum* | 5 |
| *Gorilla gorilla gorilla* | 5 |
| *Ipomoea batatas* | 5 |
| *Klebsiella pneumoniae* | 5 |
| *Loa loa* | 5 |
| *Lodderomyces elongisporus NRRL YB-4239* | 5 |
| *Loxodonta africana* | 5 |
| *Marssonina brunnea f. sp. 'multigermtubi' MB_m1* | 5 |
| *Monodelphis domestica* | 5 |
| *Myotis lucifugus* | 5 |
| *Ornithorhynchus anatinus* | 5 |
| *Oryctolagus cuniculus* | 5 |
| *Ovis aries* | 5 |
| *Pan troglodytes* | 5 |
| *Pectinophora gossypiella* | 5 |
| *Phaeosphaeria nodorum SN15* | 5 |
| *Phenacoccus solenopsis* | 5 |
| *piggyBac helper plasmid pBlu-uTp* | 5 |
| *Plasmodium yoelii yoelii 17XNL* | 5 |
| *Pseudogymnoascus destructans 20631-21* | 5 |
| *Rhizophagus irregularis DAOM 181602* | 5 |
| *Sodalis sp. HS1* | 5 |
| *Sorghum bicolor* | 5 |
| *Streptococcus anginosus* | 5 |
| *Trichosporon asahii var. asahii CBS 8904* | 5 |
| *Wolbachia endosymbiont of Drosophila ananassae* | 5 |
| *acyl-carrier-protein* | 0 |
| *Amborella trichopoda* | 4 |
| *Aspergillus niger ATCC 1015* | 4 |
| *Aspergillus niger CBS 513.88* | 4 |
| *Bactrocera tryoni* | 4 |
| *Beauveria bassiana ARSEF 2860* | 4 |
| *Beta vulgaris subsp. vulgaris* | 4 |
| *Byssochlamys spectabilis No. 5* | 4 |
| *Candidatus Regiella insecticola* | 4 |
| *Capsella rubella* | 4 |
| *Cavia porcellus* | 4 |
| *Cercopis vulnerata* | 4 |
| *Chinchilla lanigera* | 4 |
| *Cladosporium cladosporioides* | 4 |
| *Cucumis sativus* | 4 |
| *delta proteobacterium BABL1* | 4 |
| *Dictyostelium discoideum AX4* | 4 |
| *Echinococcus granulosus* | 4 |
| *Endocarpon pusillum Z07020* | 4 |
| *Entamoeba histolytica HM-1:IMSS* | 4 |
| *Entamoeba invadens IP1* | 4 |
| *Escherichia sp. 3_2_53FAA* | 4 |
| *Exophiala dermatitidis NIH/UT8656* | 4 |
| *Falco peregrinus* | 4 |
| *Felis catus* | 4 |
| *Fusobacterium mortiferum* | 4 |
| *GDP-forming* | 4 |
| *Glarea lozoyensis ATCC 20868* | 4 |
| *Gryllus bimaculatus nudivirus* | 4 |
| *Hodotermopsis sjostedti* | 4 |
| *Leptonychotes weddellii* | 4 |
| *Leuconostoc sp. DORA_2* | 4 |
| *Manduca sexta* | 4 |
| *Mayetiola destructor* | 4 |
| *Moniliophthora perniciosa FA553* | 4 |
| *Nephotettix cincticeps* | 4 |
| *Octodon degus* | 4 |
| *Oikopleura dioica* | 4 |
| *Ophiocordyceps sinensis CO18* | 4 |
| *Ophiophagus hannah* | 4 |
| *Oryctes rhinoceros virus* | 4 |
| *Oryza sativa Indica Group* | 4 |
| *Otolemur garnettii* | 4 |
| *Paracoccidioides brasiliensis Pb18* | 4 |
| *peanut chlorotic fan-spot virus* | 4 |
| *Penicillium chrysogenum Wisconsin 54-1255* | 4 |
| *Perkinsus marinus ATCC 50983* | 4 |
| *Picea sitchensis* | 4 |
| *Pisum sativum* | 4 |
| *Plutella xylostella* | 4 |
| *Pseudopodoces humilis* | 4 |
| *Rhodospirillum photometricum DSM 122* | 4 |
| *Ricinus communis* | 4 |
| *Saimiri boliviensis boliviensis* | 4 |
| *Sclerotinia borealis F-4157* | 4 |
| *Setaria italica* | 4 |
| *Sitobion avenae* | 4 |
| *Sogatella furcifera* | 4 |
| *Solanum tuberosum* | 4 |
| *Talaromyces stipitatus ATCC 10500* | 4 |
| *Tenebrio molitor* | 4 |
| *Tetraodon nigroviridis* | 4 |
| *Treponema denticola* | 4 |
| *Treponema vincentii* | 4 |
| *Trichoderma reesei QM6a* | 4 |
| *Trichoderma virens Gv29-8* | 4 |
| *Trypanosoma cruzi strain CL Brener* | 4 |
| *UDP-forming* | 4 |
| *Vanderwaltozyma polyspora DSM 70294* | 4 |
| *Zymoseptoria tritici* | 4 |
| *+* | 0 |
| *Acanthochelys spixii* | 0 |
| *Actinomyces odontolyticus* | 0 |
| *Ajellomyces dermatitidis SLH14081* | 3 |
| *Alligator mississippiensis* | 3 |
| *Ascaris suum* | 3 |
| *Aspergillus niger* | 3 |
| *Aspidiotus nerii* | 3 |
| *Auricularia delicata TFB-10046 SS5* | 3 |
| *Bacillus cereus* | 3 |
| *Blumeria graminis f. sp. hordei DH14* | 3 |
| *Botryotinia fuckeliana B05.10* | 3 |
| *Burkholderia multivorans ATCC 17616* | 3 |
| *Caligus rogercresseyi* | 3 |
| *Callithrix jacchus* | 3 |
| *Candidatus Entotheonella sp. TSY2* | 3 |
| *Candidatus Tremblaya phenacola PAVE* | 3 |
| *Chilo suppressalis* | 3 |
| *Cladophialophora carrionii CBS 160.54* | 3 |
| *Colletotrichum orbiculare MAFF 240422* | 3 |
| *Cyphellophora europaea CBS 101466* | 3 |
| *Echinops telfairi* | 3 |
| *Expression vector pBlTAL* | 3 |
| *Fusarium oxysporum Fo47* | 3 |
| *Geospiza fortis* | 3 |
| *Gossypium herbaceum* | 3 |
| *Heliothis virescens* | 3 |
| *Homalodisca vitripennis* | 3 |
| *Leptosphaeria maculans JN3* | 3 |
| *Leptotrichia hofstadii* | 3 |
| *Locusta migratoria manilensis* | 3 |
| *Lymnaea stagnalis* | 3 |
| *Macaca mulatta* | 3 |
| *Melanococcus albizziae* | 3 |
| *Meleagris gallopavo* | 3 |
| *Melitaea cinxia* | 3 |
| *Metarhizium acridum CQMa 102* | 3 |
| *Mucor circinelloides f. circinelloides 1006PhL* | 3 |
| *N-oxide-forming* | 3 |
| *Odobenus rosmarus divergens* | 3 |
| *Phanerochaete chrysosporium RP-78* | 3 |
| *Pinus koraiensis* | 3 |
| *Podospora anserina S mat+* | 3 |
| *Polysphondylium pallidum PN500* | 3 |
| *Protortonia cacti* | 3 |
| *Pseudozyma sp. GHG001* | 3 |
| *Punctularia strigosozonata HHB-11173 SS5* | 3 |
| *Pyrenophora teres f. teres 0-1* | 3 |
| *Reticulomyxa filosa* | 3 |
| *Rhizoctonia solani AG-1 IB* | 3 |
| *Salmo salar* | 3 |
| *Salpingoeca rosetta* | 3 |
| *Sclerotinia sclerotiorum 1980 UF-70* | 3 |
| *Setosphaeria turcica Et28A* | 3 |
| *Spinacia oleracea* | 3 |
| *Staphylococcus aureus* | 3 |
| *Staphylococcus epidermidis* | 3 |
| *Streptococcus iniae* | 3 |
| *Streptomyces sp. AA4* | 3 |
| *Tetrapisispora phaffii CBS 4417* | 3 |
| *Thermobia domestica* | 3 |
| *Trypanosoma congolense IL3000* | 3 |
| *Trypanosoma vivax Y486* | 3 |
| *Verticillium dahliae* | 3 |
| *Vibrio cholerae* | 3 |
| *Zonotrichia albicollis* | 3 |
| *Zygosaccharomyces rouxii* | 3 |
| *Acinetobacter towneri* | 0 |
| *ADP-ribose* | 2 |
| *Aegilops tauschii* | 2 |
| *Agrotis ipsilon* | 2 |
| *Ajellomyces capsulatus H88* | 2 |
| *Ajellomyces dermatitidis ER-3* | 2 |
| *Aphis fabae* | 2 |
| *Arabidopsis lyrata subsp. lyrata* | 2 |
| *Aspergillus nidulans* | 2 |
| *Bathycoccus prasinos* | 2 |
| *Batrachochytrium dendrobatidis JAM81* | 2 |
| *Bipolaris sorokiniana ND90Pr* | 2 |
| *Brassica oleracea* | 2 |
| *Brucella* | 2 |
| *Brucella abortus* | 2 |
| *Bryonia dioica* | 2 |
| *Burkholderia pseudomallei* | 2 |
| *Caenorhabditis elegans* | 2 |
| *Calditerrivibrio nitroreducens DSM 19672* | 2 |
| *Caligus clemensi* | 2 |
| *candidate division SR1 bacterium RAAC1_SR1_1* | 2 |
| *Candidatus Amoebophilus asiaticus 5a2* | 2 |
| *Candidatus Magnetoglobus multicellularis str. Araruama* | 2 |
| *Ceratitis rosa* | 2 |
| *Cimex lectularius* | 2 |
| *Claviceps purpurea 20.1* | 2 |
| *Clostridium butyricum DORA_1* | 2 |
| *Clostridium pasteurianum BC1* | 2 |
| *Clostridium sp. ASF502* | 2 |
| *Conocephalum conicum* | 2 |
| *Corynebacterium efficiens YS-314* | 2 |
| *Cryptocercus punctulatus* | 2 |
| *Cryptococcus neoformans* | 2 |
| *Cryptococcus neoformans var. neoformans B-3501A* | 2 |
| *Cryptotympana facialis* | 2 |
| *Ctenocephalides felis* | 2 |
| *Culex pipiens densovirus* | 2 |
| *Culicoides sonorensis* | 2 |
| *Cyanothece sp. CCY0110* | 2 |
| *decarboxylating* | 2 |
| *Dianthus caryophyllus* | 2 |
| *Diploptera punctata* | 2 |
| *Drosophila teissieri* | 2 |
| *Edhazardia aedis USNM 41457* | 2 |
| *Enterococcus pallens* | 2 |
| *Eriocheir sinensis* | 2 |
| *Eutypa lata UCREL1* | 2 |
| *Fragaria vesca subsp. vesca* | 2 |
| *Fusobacterium sp. CM22* | 2 |
| *Fusobacterium ulcerans* | 2 |
| *Halomonas titanicae* | 2 |
| *Helicoverpa zea* | 2 |
| *Heliobacterium modesticaldum Ice1* | 2 |
| *Human immunodeficiency virus 1* | 2 |
| *Johnston Atoll virus* | 2 |
| *Lactobacillus rhamnosus GG* | 2 |
| *Lampyris turkestanicus* | 2 |
| *Leishmania braziliensis MHOM/BR/75/M2904* | 2 |
| *Leishmania major strain Friedlin* | 2 |
| *Lepeophtheirus salmonis* | 2 |
| *Leptopilina clavipes* | 2 |
| *Leptotrichia wadei* | 2 |
| *Lethocerus indicus* | 2 |
| *Macrosiphum euphorbiae* | 2 |
| *Macrotermes barneyi* | 2 |
| *Magnaporthe oryzae 70-15* | 2 |
| *Magnetospirillum gryphiswaldense MSR-1 v2* | 2 |
| *Methylomicrobium alcaliphilum 20Z* | 2 |
| *Micromonas pusilla CCMP1545* | 2 |
| *Mn* | 2 |
| *Monosiga brevicollis MX1* | 2 |
| *Mycoplasma penetrans HF-2* | 2 |
| *NADP+* | 2 |
| *Neosartorya fischeri NRRL 181* | 2 |
| *Nicotiana tabacum* | 2 |
| *Papilio dardanus* | 2 |
| *Papio anubis* | 2 |
| *Paracoccidioides brasiliensis Pb03* | 2 |
| *Paracoccus nothofagicola* | 2 |
| *Pediococcus pentosaceus ATCC 25745* | 2 |
| *Penicillium digitatum PHI26* | 2 |
| *Phenacoccus solani* | 2 |
| *Phyllostachys edulis* | 2 |
| *Physcomitrella patens* | 2 |
| *Phytolacca acinosa* | 2 |
| *Plasmodium chabaudi chabaudi* | 2 |
| *Plasmodium falciparum 3D7* | 2 |
| *Pongo abelii* | 2 |
| *Procambarus clarkii* | 2 |
| *Propionibacterium acnes* | 2 |
| *Prunus persica* | 2 |
| *Pseudoalteromonas agarivorans* | 2 |
| *Pseudoplusia includens* | 2 |
| *Rhopalosiphum padi* | 2 |
| *Rhynchosciara americana* | 2 |
| *Rhynchosporium commune* | 2 |
| *Roseburia intestinalis XB6B4* | 2 |
| *Salsola komarovii* | 2 |
| *Sclerotinia sclerotiorum 1980* | 2 |
| *Silene latifolia* | 2 |
| *Solanum chilense* | 2 |
| *Solanum demissum* | 2 |
| *Solanum lycopersicum* | 2 |
| *Sordaria macrospora k-hell* | 2 |
| *Sorex araneus* | 2 |
| *Spodoptera littoralis* | 2 |
| *starch* | 2 |
| *Streptococcus agalactiae* | 2 |
| *Streptococcus parasanguinis* | 2 |
| *Streptococcus pneumoniae* | 2 |
| *Streptococcus salivarius* | 2 |
| *Streptococcus vestibularis* | 2 |
| *Streptomyces* | 2 |
| *Streptomyces sp. e14* | 2 |
| *Streptomyces sp. SPB78* | 2 |
| *Taeniopygia guttata* | 2 |
| *Tetrapisispora blattae CBS 6284* | 2 |
| *Thermosipho africanus TCF52B* | 2 |
| *Togninia minima UCRPA7* | 2 |
| *Toxoplasma gondii ME49* | 2 |
| *Tremella fuciformis* | 2 |
| *Treponema phagedenis* | 2 |
| *Trichoderma atroviride IMI 206040* | 2 |
| *Triticum urartu* | 2 |
| *Tursiops truncatus* | 2 |
| *uncultured beta proteobacterium HF0130_04F21* | 2 |
| *uncultured virus* | 2 |
| *Verticillium alfalfae VaMs.102* | 2 |
| *Vicugna pacos* | 2 |
| *Wolbachia endosymbiont of Drosophila simulans wHa* | 2 |
| *Acanthamoeba castellanii str. Neff* | 0 |
| *Acaulospora laevis* | 0 |
| *Acer pseudoplatanus* | 0 |
| *Acheta domesticus* | 0 |
| *Acinetobacter baumannii 1046051* | 0 |
| *Acinetobacter baumannii 136706* | 0 |
| *Acinetobacter baumannii CI78* | 0 |
| *Acinetobacter sp. P8-3-8* | 0 |
| *Actinomyces sp. oral taxon 181* | 0 |
| *acylating* | 0 |
| *Adelphocoris suturalis* | 1 |
| *Agaricus bisporus var. bisporus* | 1 |
| *Agaricus bisporus var. bisporus H97* | 1 |
| *Agrotis exclamationis* | 1 |
| *Ajellomyces capsulatus G186AR* | 1 |
| *Ajellomyces dermatitidis ATCC 18188* | 1 |
| *Ajellomyces dermatitidis ATCC 26199* | 1 |
| *Albugo laibachii Nc14* | 1 |
| *Alistipes sp. CAG:831* | 1 |
| *Alnus glutinosa* | 1 |
| *Alternaria cirsinoxia* | 1 |
| *Amaranthus cruentus/Amaranthus hypocondriacus mixed library* | 1 |
| *Amaranthus retroflexus* | 1 |
| *Anasa tristis* | 1 |
| *Anisopteromalus calandrae* | 1 |
| *Anopheles albimanus* | 1 |
| *Antheraea pernyi iflavirus* | 1 |
| *Antheraea yamamai* | 1 |
| *Aphanomyces astaci* | 1 |
| *Aphid lethal paralysis virus* | 1 |
| *Aphis medicaginis* | 1 |
| *Aphis nerii* | 1 |
| *Aphonopelma sp. WDB-1998* | 1 |
| *Apodemus latronum* | 1 |
| *Apodemus peninsulae* | 1 |
| *Apolygus lucorum* | 1 |
| *Arachis hypogaea* | 1 |
| *Artemia sinica* | 1 |
| *Artemia sp.* | 1 |
| *Arthrobotrys oligospora ATCC 24927* | 1 |
| *Ascidia sydneiensis samea* | 1 |
| *Ascosphaera torchioi* | 1 |
| *Aspergillus flavus* | 1 |
| *Aspergillus kawachii IFO 4308* | 1 |
| *asymmetrical* | 1 |
| *Athalia rosae* | 1 |
| *ATP* | 1 |
| *Atriplex nummularia* | 1 |
| *Aurantimonas manganoxydans* | 1 |
| *Azospirillum sp. CAG:260* | 1 |
| *Azumapecten farreri* | 1 |
| *Bacillus acidiproducens* | 1 |
| *Bacillus licheniformis* | 1 |
| *Bacillus phage G* | 1 |
| *Bacillus thuringiensis YBT-1518* | 1 |
| *Bacillus weihenstephanensis NBRC 101238 = DSM 11821* | 1 |
| *Bacteriophage APSE-4* | 1 |
| *Bacteroides nordii* | 1 |
| *Bacteroides salyersiae* | 1 |
| *Bacteroides sp. 2_2_4* | 1 |
| *Bacteroides sp. CAG:189* | 1 |
| *Beggiatoa sp. PS* | 1 |
| *Bifidobacterium bifidum* | 1 |
| *Bifidobacterium longum* | 1 |
| *Bipolaris maydis C5* | 1 |
| *Blaberus discoidalis* | 1 |
| *Blumeria graminis f. sp. tritici 96224* | 1 |
| *Boisea trivittata* | 1 |
| *Bombyx mandarina* | 1 |
| *Bordetella bronchiseptica MO149* | 1 |
| *Brachypodium distachyon* | 1 |
| *Brachyspira intermedia PWS/A* | 1 |
| *Branchiostoma californiense* | 1 |
| *Brassica napus* | 1 |
| *Brassica oleracea var. botrytis* | 1 |
| *Bruguiera gymnorhiza* | 1 |
| *Buchnera aphidicola str. Bp (Baizongia pistaciae)* | 1 |
| *Buchnera aphidicola str. USDA (Myzus persicae)* | 1 |
| *Burkholderia lata* | 1 |
| *Burkholderia sp. CCGE1002* | 1 |
| *Burkholderia thailandensis MSMB121* | 1 |
| *Burkholderiales* | 1 |
| *Bursaphelenchus xylophilus* | 1 |
| *Butyrivibrio sp. CAG:318* | 1 |
| *Buxus microphylla* | 1 |
| *Callosobruchus maculatus* | 1 |
| *Camellia sinensis* | 1 |
| *Candida albicans WO-1* | 1 |
| *Candida dubliniensis CD36* | 1 |
| *Candida maltosa Xu316* | 1 |
| *Candidatus Blochmannia floridanus* | 1 |
| *Candidatus Entotheonella sp. TSY1* | 1 |
| *Canis lupus* | 1 |
| *Canis sp.* | 1 |
| *Capparis hastata* | 1 |
| *Carassius auratus* | 1 |
| *Cardinium endosymbiont cEper1 of Encarsia pergandiella* | 1 |
| *Catocyclotis adelina* | 1 |
| *Ceratotherium simum simum* | 1 |
| *Ceriporiopsis subvermispora B* | 1 |
| *Chaetomium globosum CBS 148.51* | 1 |
| *Chaetomium thermophilum var. thermophilum DSM 1495* | 1 |
| *Chironomus tentans* | 1 |
| *Chitinophaga pinensis DSM 2588* | 1 |
| *Chondrus crispus* | 1 |
| *Chrysomela tremula* | 1 |
| *Citrus hybrid cultivar* | 1 |
| *Clavispora lusitaniae ATCC 42720* | 1 |
| *Cloning vector pFBneo* | 1 |
| *Clostridium botulinum E3 str. Alaska E43* | 1 |
| *Clostridium carboxidivorans* | 1 |
| *Clostridium leptum* | 1 |
| *Clostridium pasteurianum* | 1 |
| *Clostridium sp. CAG:253* | 1 |
| *Clostridium sp. CAG:43* | 1 |
| *Coccidioides posadasii str. Silveira* | 1 |
| *Coenonympha glycerion* | 1 |
| *Colletotrichum gloeosporioides Cg-14* | 1 |
| *Colletotrichum graminicola M1.001* | 1 |
| *Colletotrichum sp. zzgmzg1* | 1 |
| *Coprinopsis cinerea okayama7#130* | 1 |
| *Coprococcus comes* | 1 |
| *Coprococcus eutactus* | 1 |
| *Corallorhiza maculata var. mexicana* | 1 |
| *Cordyceps militaris CM01* | 1 |
| *Corynebacterium mastitidis* | 1 |
| *Corynebacterium pseudogenitalium* | 1 |
| *Cotesia congregata* | 1 |
| *Cotesia sesamiae Mombasa bracovirus* | 1 |
| *Cotesia vestalis bracovirus* | 1 |
| *Cronobacter turicensis z3032* | 1 |
| *Ctenoplusia agnata* | 1 |
| *Curtobacterium sp. B8* | 1 |
| *Cylindrospermopsis raciborskii* | 1 |
| *Cyprinus carpio* | 1 |
| *Dactylellina haptotyla CBS 200.50* | 1 |
| *Dastarcus helophoroides* | 1 |
| *Datura stramonium* | 1 |
| *Debaryomyces hansenii CBS767* | 1 |
| *Deformed wing virus* | 1 |
| *Deinococcus radiodurans R1* | 1 |
| *Dekkera bruxellensis AWRI1499* | 1 |
| *Densovirus SC1065* | 1 |
| *Desulfococcus multivorans* | 1 |
| *Desulfovibrio magneticus RS-1* | 1 |
| *Diadromus pulchellus ascovirus 4a* | 1 |
| *Dianemobius nigrofasciatus* | 1 |
| *Dictyostelium purpureum* | 1 |
| *Discocelis tigrina* | 1 |
| *Diuraphis noxia* | 1 |
| *Drosophila buzzatii* | 1 |
| *Duganella zoogloeoides* | 1 |
| *Eimeria brunetti* | 1 |
| *Elizabethkingia anophelis* | 1 |
| *Emiliania huxleyi virus 18* | 1 |
| *Endocarpon pusillum* | 1 |
| *Endozoicomonas elysicola* | 1 |
| *Enterobacter sp. MGH 24* | 1 |
| *Enterobacteria phage IME10* | 1 |
| *Enterococcus faecalis* | 1 |
| *Enterococcus sulfureus* | 1 |
| *Epidermophyton floccosum* | 1 |
| *Episyrphus balteatus* | 1 |
| *Eptatretus stoutii* | 1 |
| *Eremothecium cymbalariae DBVPG#7215* | 1 |
| *Erwinia amylovora* | 1 |
| *Erysipelotrichaceae bacterium 2_2_44A* | 1 |
| *Escherichia coli APEC O1* | 1 |
| *Escherichia coli BW2952* | 1 |
| *Escherichia coli IS25* | 1 |
| *Escherichia coli O55:H7 str. RM12579* | 1 |
| *Escherichia coli SE11* | 1 |
| *Escherichia coli UTI89* | 1 |
| *Eubacterium rectale DSM 17629* | 1 |
| *Eucommia ulmoides* | 1 |
| *Euplokamis dunlapae* | 1 |
| *FAD, quinone* | 1 |
| *Festuca ovina* | 1 |
| *Fibroporia radiculosa* | 1 |
| *Fusarium oxysporum* | 1 |
| *Fusarium oxysporum f. sp. lycopersici MN25* | 1 |
| *Fusobacterium necrophorum* | 1 |
| *Fusobacterium sp. CM1* | 1 |
| *Fusobacterium sp. OBRC1* | 1 |
| *Fusobacterium varium* | 1 |
| *Gaeumannomyces graminis var. tritici R3-111a-1* | 1 |
| *Galdieria sulphuraria* | 1 |
| *Gampsocleis gratiosa* | 1 |
| *Georissus sp. APV-2005* | 1 |
| *Gloeophyllum trabeum ATCC 11539* | 1 |
| *Gluconacetobacter xylinus E25* | 1 |
| *Gossypium darwinii* | 1 |
| *Grosmannia clavigera kw1407* | 1 |
| *Gryllotalpa orientalis* | 1 |
| *Gryllus firmus* | 1 |
| *Gryllus pennsylvanicus* | 1 |
| *Haemophilus parainfluenzae* | 1 |
| *Haemophilus parainfluenzae T3T1* | 1 |
| *Halanaerobium praevalens DSM 2228* | 1 |
| *Haliotis diversicolor supertexta* | 1 |
| *Helianthus annuus* | 1 |
| *Helianthus tuberosus* | 1 |
| *Helicobacter felis ATCC 49179* | 1 |
| *Helicobacter pylori* | 1 |
| *Heliconius erato* | 1 |
| *Heliconius melpomene* | 1 |
| *Heliococcus bohemicus* | 1 |
| *Hemiselmis andersenii* | 1 |
| *Hevea brasiliensis* | 1 |
| *Hydrogenobaculum sp. Y04AAS1* | 1 |
| *Icacina mannii* | 1 |
| *Ichthyophthirius multifiliis* | 1 |
| *Ictidomys tridecemlineatus* | 1 |
| *Ilyanassa obsoleta* | 1 |
| *Invertebrate iridescent virus 6* | 1 |
| *Ips typographus* | 1 |
| *Jaculus jaculus* | 1 |
| *Janiodes laverna* | 1 |
| *Jatropha curcas* | 1 |
| *Karlodinium veneficum* | 1 |
| *Kwoniella heveanensis* | 1 |
| *Lachnospiraceae bacterium 3_1_57FAA_CT1* | 1 |
| *Lachnospiraceae bacterium A4* | 1 |
| *Lachnus roboris* | 1 |
| *Lactobacillus casei LC2W* | 1 |
| *Lactobacillus jensenii* | 1 |
| *Lactococcus lactis* | 1 |
| *Lagenaria siceraria* | 1 |
| *Latrodectus hesperus* | 1 |
| *Leishmania mexicana MHOM/GT/2001/U1103* | 1 |
| *Lens culinaris subsp. culinaris* | 1 |
| *Lepismachilis sp. VK-2004* | 1 |
| *Leptinotarsa decemlineata* | 1 |
| *Leptotrichia buccalis C-1013-b* | 1 |
| *Leptotrichia sp. oral taxon 225* | 1 |
| *Leptotrichia sp. oral taxon 879* | 1 |
| *Lestes congener* | 1 |
| *Leucaena leucocephala* | 1 |
| *Liberibacter crescens BT-1* | 1 |
| *Lingulodinium polyedrum* | 1 |
| *lipoamide* | 1 |
| *Liposcelis decolor* | 1 |
| *Litchi chinensis* | 1 |
| *Lonomia obliqua* | 1 |
| *Lycoris longituba* | 1 |
| *Lygus lineolaris* | 1 |
| *Macrobrachium nipponense* | 1 |
| *Magnolia kobus* | 1 |
| *Manihot esculenta* | 1 |
| *Marine Group I thaumarchaeote SCGC AB-629-I23* | 1 |
| *Marinobacter hydrocarbonoclasticus ATCC 49840* | 1 |
| *Marsupenaeus japonicus* | 1 |
| *Marvinbryantia formatexigens* | 1 |
| *Megoura crassicauda* | 1 |
| *Melampsora larici-populina 98AG31* | 1 |
| *Melopsittacus undulatus* | 1 |
| *Methanolobus tindarius* | 1 |
| *Methylobacillus flagellatus KT* | 1 |
| *Methylotenera* | 1 |
| *Methylovulum miyakonense* | 1 |
| *Millerozyma farinosa CBS 7064* | 1 |
| *Millettia pinnata* | 1 |
| *Mirabilis jalapa* | 1 |
| *Miscanthicoccus miscanthi* | 1 |
| *Mizuhopecten yessoensis* | 1 |
| *Mortierella alpina* | 1 |
| *Muntiacus muntjak* | 1 |
| *Mus caroli* | 1 |
| *Mus musculus domesticus* | 1 |
| *Mus musculus musculus* | 1 |
| *Mus sp.* | 1 |
| *Musa acuminata* | 1 |
| *Mycobacterium tuberculosis* | 1 |
| *Mycoplasma arthritidis 158L3-1* | 1 |
| *Mythimna separata entomopoxvirus 'L'* | 1 |
| *NADP* | 1 |
| *NADP(+)* | 1 |
| *Nannochloropsis gaditana CCMP526* | 1 |
| *Nasutitermes fumigatus* | 1 |
| *Natranaerobius thermophilus JW/NM-WN-LF* | 1 |
| *Nectria sp. CBS 478.75* | 1 |
| *Negev virus* | 1 |
| *Nematocida parisii ERTm3* | 1 |
| *Neurospora tetrasperma FGSC 2508* | 1 |
| *Neurospora tetrasperma FGSC 2509* | 1 |
| *Nicotiana sylvestris* | 1 |
| *Nocardiopsis kunsanensis* | 1 |
| *Nosema apis BRL 01* | 1 |
| *Nyctotherus ovalis* | 1 |
| *Nylanderia nr. pubens LZ-2010* | 1 |
| *Ochlerotatus atropalpus* | 1 |
| *Ochotona hyperborea* | 1 |
| *Oenothera berteroana* | 1 |
| *Omphisa fuscidentalis* | 1 |
| *Oncometopia nigricans* | 1 |
| *Oncopeltus fasciatus* | 1 |
| *Oncorhynchus masou* | 1 |
| *Oncorhynchus mykiss* | 1 |
| *Ophiocordyceps unilateralis* | 1 |
| *Ophiostoma piceae UAMH 11346* | 1 |
| *Orchesella cincta* | 1 |
| *Orcinus orca* | 1 |
| *Orenia marismortui* | 1 |
| *Oreta pulchripes* | 1 |
| *Orgyia leucostigma NPV* | 1 |
| *Pan paniscus* | 1 |
| *Parabacteroides goldsteinii* | 1 |
| *Paracoccidioides sp. 'lutzii' Pb01* | 1 |
| *Parafronurus youi* | 1 |
| *Paramecium tetraurelia strain d4-2* | 1 |
| *Paratlanticus ussuriensis* | 1 |
| *Pediococcus pentosaceus SL4* | 1 |
| *Pelobacter propionicus DSM 2379* | 1 |
| *Penicillium oxalicum 114-2* | 1 |
| *Penicillium roqueforti* | 1 |
| *Pepper cryptic virus 2* | 1 |
| *Petromyzon marinus* | 1 |
| *Petroselinum crispum* | 1 |
| *Phalaenopsis aphrodite subsp. formosana* | 1 |
| *Phanerochaete carnosa HHB-10118-sp* | 1 |
| *Phanerotoma flava* | 1 |
| *Phascolarctobacterium sp. CAG:207* | 1 |
| *Phialocephala subalpina* | 1 |
| *Phillyrea latifolia* | 1 |
| *Phlebotomus ariasi* | 1 |
| *Phragmatopoma californica* | 1 |
| *Phyllobolus splendens* | 1 |
| *Phytolacca americana* | 1 |
| *Phytophthora infestans T30-4* | 1 |
| *Phytophthora sojae* | 1 |
| *Piriformospora indica DSM 11827* | 1 |
| *Placospermum coriaceum* | 1 |
| *Plasmodium falciparum Palo Alto/Uganda* | 1 |
| *Plasmodium vinckei petteri* | 1 |
| *Platynereis dumerilii* | 1 |
| *Plautia stali* | 1 |
| *Plectus acuminatus* | 1 |
| *Populus tremula x Populus tremuloides* | 1 |
| *Portulaca oleracea* | 1 |
| *Praomys tullbergi* | 1 |
| *Prevotella sp. CAG:732* | 1 |
| *Proteus mirabilis HI4320* | 1 |
| *Providencia alcalifaciens PAL-3* | 1 |
| *Pseudoalteromonas tunicata* | 1 |
| *Pseudomonas putida HB3267* | 1 |
| *Pterocomma pilosum* | 1 |
| *Pyronema omphalodes CBS 100304* | 1 |
| *Pyropia yezoensis* | 1 |
| *Pyruvate dehydrogenase* | 1 |
| *Quaranfil virus* | 1 |
| *Ralstonia solanacearum Po82* | 1 |
| *Rhipicephalus microplus* | 1 |
| *Rhizobium sp.* | 1 |
| *Rhyparobia maderae* | 1 |
| *Rickettsia prowazekii* | 1 |
| *Rickettsiella grylli* | 1 |
| *Rosa rugosa* | 1 |
| *Roseobacter sp. AzwK-3b* | 1 |
| *Saccharomyces cerevisiae* | 1 |
| *Saccharomycetaceae sp. 'Ashbya aceri'* | 1 |
| *Salicornia europaea* | 1 |
| *Salmonella enterica subsp. enterica serovar Gallinarum/pullorum str. FCAV198* | 1 |
| *Salvia miltiorrhiza* | 1 |
| *Saprolegnia diclina VS20* | 1 |
| *Sarcoptes scabiei type hominis* | 1 |
| *Scheffersomyces stipitis CBS 6054* | 1 |
| *Schiedea trinervis* | 1 |
| *Schizolachnus orientalis* | 1 |
| *Schizosaccharomyces cryophilus OY26* | 1 |
| *secondary endosymbiont of Ctenarytaina eucalypti* | 1 |
| *Selaginella moellendorffii* | 1 |
| *Serpula lacrymans var. lacrymans S7.3* | 1 |
| *Serratia marcescens WW4* | 1 |
| *Serratia symbiotica str. 'Cinara cedri'* | 1 |
| *Shigella flexneri 5 str. 8401* | 1 |
| *Simian immunodeficiency virus* | 1 |
| *Sodalis glossinidius str. 'morsitans'* | 1 |
| *Solanum habrochaites* | 1 |
| *Solanum melongena* | 1 |
| *Spathaspora passalidarum NRRL Y-27907* | 1 |
| *Sphaerius sp. APV-2005* | 1 |
| *Spodoptera exigua* | 1 |
| *Spodoptera frugiperda* | 1 |
| *Spodoptera litura* | 1 |
| *Sporolactobacillus inulinus* | 1 |
| *Spraguea lophii 42_110* | 1 |
| *Staphylococcus aureus M0125* | 1 |
| *Staphylococcus capitis* | 1 |
| *Staphylococcus vitulinus* | 1 |
| *Streptococcus anginosus DORA_7* | 1 |
| *Streptococcus pyogenes* | 1 |
| *Streptomyces coelicoflavus* | 1 |
| *Streptomyces sp. C* | 1 |
| *Strongyloides papillosus* | 1 |
| *Suaeda japonica* | 1 |
| *Succinivibrionaceae bacterium WG-1* | 1 |
| *Sulfurihydrogenibium azorense Az-Fu1* | 1 |
| *Sulfurihydrogenibium yellowstonense* | 1 |
| *Sutterella wadsworthensis CAG:135* | 1 |
| *Sycon ciliatum* | 1 |
| *Talaromyces marneffei ATCC 18224* | 1 |
| *Taylorella asinigenitalis 14/45* | 1 |
| *Teratosphaeria pseudoeucalypti* | 1 |
| *Tetraneura sp. ZMIOZ 22400* | 1 |
| *Tetranychus cinnabarinus* | 1 |
| *Tetrasphaera elongata* | 1 |
| *Theileria parva strain Muguga* | 1 |
| *Thermosipho melanesiensis BI429* | 1 |
| *Theromyzon rude* | 1 |
| *Tjuloc virus* | 1 |
| *Torulaspora delbrueckii* | 1 |
| *Toxorhynchites brevipalpis* | 1 |
| *Trichechus manatus latirostris* | 1 |
| *Trichophyton tonsurans CBS 112818* | 1 |
| *Trichuris suis* | 1 |
| *Tripneustes gratilla* | 1 |
| *Triticum turgidum* | 1 |
| *Tuberaphis coreana* | 1 |
| *Tuberolachnus salignus* | 1 |
| *uncultured bacterium* | 1 |
| *uncultured bacterium A1Q1_fos_4* | 1 |
| *uncultured bacterium CSL142* | 1 |
| *uncultured beta proteobacterium CBNPD1 BAC clone 578* | 1 |
| *uncultured Desulfobacterium sp.* | 1 |
| *uncultured fungus* | 1 |
| *uncultured Verrucomicrobiales bacterium HF0200_39L05* | 1 |
| *Vryburgia amaryllidis* | 1 |
| *Wallemia ichthyophaga EXF-994* | 1 |
| *Weissella koreensis KACC 15510* | 1 |
| *Wolbachia endosymbiont strain TRS of Brugia malayi* | 1 |
| *Wolffia arrhiza* | 1 |
| *Wuchereria bancrofti* | 1 |
| *Xenopsylla cheopis* | 1 |
| *Xenorhabdus szentirmaii DSM 16338* | 1 |
| *Xerophyta humilis* | 1 |
| *Yarrowia lipolytica* | 1 |
| *Zantedeschia aethiopica* | 1 |
| *Zea mays subsp. mexicana* | 1 |
| *Zootermopsis nevadensis* | 1 |

**Table S3:** Biological Process category annotation by Gene Ontology for differentially expressed transcripts (DET) for pair-wise comparisons between developmental stages of *P. solenopsis* by AgriGO Database.

| **Compared Samples** | **Gene Ontology Term** | **Transcripts** |
| --- | --- | --- |
|  | Metabolic process | 198 |
|  | Cellular process | 126 |
|  | Metabolic process | 143 |
|  | Primary metabolic process | 153 |
|  | Cellular metabolic process | 123 |
|  | Macromolecule metabolic process | 153 |
|  | Gene expression | 37 |
|  | Protein metabolic process | 61 |
| **EggI Vs Second Instar** | Biological regulation | 38 |
|  | Cellular protein metabolic process | 30 |
|  | Regulation of biological process | 34 |
|  | Regulation of cellular process | 82 |
|  | RNA metabolic process | 25 |
|  | Cellular biosynthetic process | 74 |
|  | Nitrogen compound metabolic process | 104 |
|  | Transcription | 27 |
|  | Macromolecule modification | 22 |
|  | Protein modification process | 20 |
|  | Regulation of metabolic process | 29 |
|  | cellular process | 51 |
|  | metabolic process | 56 |
|  | primary metabolic process | 64 |
|  | cellular metabolic process | 62 |
|  | macromolecule metabolic process | 65 |
|  | cellular macromolecule metabolic process | 60 |
|  | gene expression | 38 |
|  | protein metabolic process | 23 |
| **Second Instar Vs Third Instar** | biological regulation | 23 |
|  | cellular protein metabolic process | 18 |
|  | regulation of biological process | 22 |
|  | regulation of cellular process | 22 |
|  | biosynthetic process | 54 |
|  | RNA metabolic process | 16 |
|  | cellular biosynthetic process | 53 |
|  | nitrogen compound metabolic process | 43 |
|  | macromolecule modification | 4 |
|  | protein modification process | 4 |
|  | regulation of metabolic process | 19 |

**Table S4:** Molecular Function category annotation by Gene Ontology for differentially expressed transcripts (DET) for pair-wise comparisons between developmental stages of *P. solenopsis* by AgriGO Database.

| **Compared Samples** | **Gene Ontology Term** | **Transcripts** |
| --- | --- | --- |
|  | binding | 158 |
|  | catalytic activity | 132 |
|  | purine nucleotide binding | 52 |
|  | nucleotide binding | 52 |
|  | ATP binding | 39 |
|  | adenyl ribonucleotide binding | 39 |
|  | hydrolase activity | 129 |
| **EggI Vs Second Instar** | transcription regulator activity | 107 |
|  | sequence-specific DNA binding | 9 |
|  | kinase activity | 16 |
|  | ion binding | 38 |
|  | cation binding | 38 |
|  | phosphotransferase activity, alcohol group as acceptor | 16 |
|  | transcription factor activity | 15 |
|  | binding | 84 |
|  | catalytic activity | 38 |
|  | purine nucleotide binding | 10 |
|  | nucleotide binding | 10 |
|  | ATP binding | 8 |
|  | adenyl ribonucleotide binding | 8 |
|  | hydrolase activity | 13 |
| **Second Instar Vs Third Instar** | DNA binding | 23 |
|  | transcription regulator activity | 19 |
|  | nucleic acid binding | 42 |
|  | sequence-specific DNA binding | 12 |
|  | kinase activity | 3 |
|  | ion binding | 12 |
|  | cation binding | 13 |
|  | phosphotransferase activity, alcohol group as acceptor | 3 |
|  | transcription factor activity | 13 |
|  | catalytic activity | 44 |
|  | purine nucleotide binding | 19 |
|  | nucleotide binding | 21 |
|  | ATP binding | 11 |
|  | adenyl ribonucleotide binding | 12 |
|  | hydrolase activity | 26 |
| **Third Vs Adult female** | DNA binding | 26 |
|  | nucleic acid binding | 45 |
|  | sequence-specific DNA binding | 7 |
|  | kinase activity | 2 |
|  | ion binding | 12 |
|  | cation binding | 12 |
|  | phosphotransferase activity, alcohol group as acceptor | 2 |
|  | transcription factor activity | 4 |

**Table S5:** Cellular Components category annotation by Gene Ontology for differentially expressed transcripts (DET) for pair-wise comparisons between developmental stages of *P. solenopsis* by AgriGO Database.

| **Compared Samples** | **Gene Ontology Term** | **Transcripts** |
| --- | --- | --- |
|  | intracellular | 78 |
|  | intracellular part | 57 |
|  | intracellular organelle | 53 |
| **EggI Vs Second Instar** | organelle | 52 |
|  | membrane | 8 |
|  | intracellular membrane-bounded organelle | 44 |
|  | cytoplasm | 13 |
|  | nucleus | 56 |
|  | cytoplasmic part | 13 |
|  | intracellular | 42 |
|  | intracellular part | 37 |
|  | intracellular organelle | 35 |
|  | membrane | 27 |
| **Second Vs Third Instar** | membrane-bounded organelle | 19 |
|  | cytoplasm | 17 |
|  | nucleus | 19 |
|  | macromolecular complex | 17 |
|  | cytoplasmic part | 15 |
|  | protein complex | 2 |
|  | intracellular | 25 |
|  | intracellular part | 17 |
|  | intracellular organelle | 17 |
|  | membrane | 34 |
| **Third Vs Adult female** | membrane-bounded organelle | 12 |
|  | cytoplasm | 6 |
|  | nucleus | 7 |
|  | macromolecular complex | 11 |
|  | cytoplasmic part | 6 |
|  | protein complex | 6 |
|  | intracellular | 123 |
|  | intracellular part | 91 |
|  | intracellular organelle | 84 |
|  | membrane | 49 |
| **EggI Vs Adult female** | intracellular membrane-bounded organelle | 68 |
|  | membrane-bounded organelle | 81 |
|  | nucleus | 61 |
|  | macromolecular complex | 29 |
|  | cytoplasmic part | 17 |
|  | protein complex | 9 |

**Table S6:** The top 10 up or down-regulated genes identified among first instar vs second instar, second instar vs third instar, and third instar vs adult female and adult female vs first instar.

| **Transcript_ID** | **Source** | **Description** | **Eggs/First Instar** | **Second** | **Third** | **Adult_Female** |
| --- | --- | --- | --- | --- | --- | --- |
| TRINITY_DN21076_c3_g5 | EggI | hypothetical protein Clole_3223 | 38915.53 | 100243.39 | 50409.68 | 2.06 |
| TRINITY_DN18416_c0_g1 | EggI | Uncharacterized | 193.26 | 921.47 | 1063.21 | 0 |
| TRINITY_DN21076_c3_g9 | EggI | Uncharacterized | 9805.03 | 22562.03 | 9952.29 | 0 |
| TRINITY_DN1310_c0_g1 | EggI | Uncharacterized | 941.68 | 1497.27 | 1250.06 | 0.05 |
| TRINITY_DN44711_c0_g1 | EggI | putative antimicrobial knottin protein Btk-4 [*Bemisia tabaci* | 187.9 | 468.75 | 503.56 | 0 |
| TRINITY_DN15648_c0_g1 | EggI | hypothetical protein BBA_03436 [*Beauveria bassiana* ARSEF 2860 | 495.91 | 578.37 | 617.26 | 0.13 |
| TRINITY_DN21076_c3_g4 | EggI | hypothetical protein Clole_3223 [*Clostridium lentocellum* DSM 5427 | 17150.45 | 40283.22 | 19195.17 | 1.24 |
| TRINITY_DN15648_c0_g2 | EggI | BBA_03436 [*Beauveria bassiana* ARSEF 2860 | 699.73 | 788.91 | 471.68 | 0.13 |
| TRINITY_DN23214_c5_g24 | EggI | serine proteinase [*Portunus trituberculatus* | 125.85 | 194.23 | 426.48 | 0.01 |
| TRINITY_DN70757_c0_g2 | EggI | Eukaryotic translation initiation factor 3  subunit, putative [*Pediculus humanus corporis* | 39.22 | 44.33 | 74.31 | 0 |
| TRINITY_DN25769_c3_g1 | EggI | hypothetical protein LOC100573291 [*Acyrthosiphon pisum* | 3.36 | 2.16 | 10.78 | 2.2 |
| TRINITY_DN16608_c0_g4 | EggI | tumor protein p63 isoform alpha 2-like [Acyrthosiphon pisum | 3.8 | 2.96 | 3.42 | 1.15 |
| TRINITY_DN21430_c0_g1 | EggI | DNA replication licensing factor MCM8, putative [*Pediculus humanus corporis* | 6.89 | 7.3 | 6.92 | 2.31 |
| TRINITY_DN18186_c1_g1 | EggI | \|hypothetical protein YQE_07182, partial [*Dendroctonus ponderosae* | 1.3 | 2.3 | 2.6 | 0.89 |
| TRINITY_DN17106_c0_g1 | EggI | Uncharacterized | 39.71 | 47.1 | 55.88 | 20.74 |
| TRINITY_DN35717_c0_g1 | EggI | hypothetical protein D910_06514 [*Dendroctonus ponderosae* | 1.19 | 0.93 | 2.99 | 1.06 |
| TRINITY_DN14127_c0_g1 | EggI | hypothetical protein YQE_09794, partial [*Dendroctonus ponderosae* | 5.14 | 4.74 | 5.67 | 2.34 |
| TRINITY_DN59607_c0_g1 | EggI | Uncharacterized | 12.97 | 7.16 | 6.53 | 2.78 |
| TRINITY_DN11825_c0_g1 | EggsI | Uncharacterized | 5.98 | 3.48 | 6.61 | 2.82 |
| TRINITY_DN5186_c0_g1 | EggI | Uncharacterized | 0 | 0.27 | 1.55 | 0.52 |
| TRINITY_DN52932_c1_g1 | Second_Instar | nonstructural polyprotein [Aphid lethal paralysis virus | 4936.43 | 1208.14 | 0.01 | 0 |
| TRINITY_DN34955_c0_g1 | Second_Instar | heat shock protein 40 [*Locusta migratoria* | 0 | 19.25 | 0 | 24.81 |
| TRINITY_DN24373_c5_g2 | Second_Instar | Uncharacterized | 8.27 | 16.21 | 0 | 0 |
| TRINITY_DN20917_c4_g1 | Second_Instar | DnaJ domain-containing protein [*Bombyx mori* | 0 | 16.13 | 0 | 9.56 |
| TRINITY_DN13458_c0_g1 | Second_Instar | \|protein C20orf121, putative [*Pediculus humanus corporis* | 6.07 | 6.36 | 0 | 3.93 |
| TRINITY_DN18031_c2_g11 | Second_Instar | PREDICTED: similar to CG3244 CG3244-PA isoform 1 [*Tribolium castaneum* | 12.19 | 10.73 | 0 | 12.95 |
| TRINITY_DN61911_c0_g2 | Second_Instar | 60S ribosomal protein L18-like [*Acyrthosiphon pisum* | 6.19 | 7.43 | 0 | 2.19 |
| TRINITY_DN27268_c0_g4 | Second_Instar | Uncharacterized | 8.51 | 4.5 | 0 | 4.25 |
| TRINITY_DN4719_c0_g5 | Second_Instar | actin-related protein [*Riptortus pedestris* | 8.77 | 2.57 | 0 | 0.03 |
| TRINITY_DN61911_c0_g6 | Second_Instar | 60S ribosomal protein L18-like [*Acyrthosiphon pisum* | 1.69 | 4.97 | 0 | 2.48 |
| TRINITY_DN2381_c0_g1 | Second_Instar | DNA mismatch repair protein Mlh1-like [*Acyrthosiphon pisum* | 2.38 | 1.6 | 0.45 | 1.66 |
| TRINITY_DN18031_c2_g2 | Second_Instar | similar to CG3244 CG3244-PA isoform 1 [*Tribolium castaneum* | 2.65 | 4.22 | 1.19 | 2.03 |
| TRINITY_DN20863_c0_g1 | Second_Instar | Uncharacterized | 0.74 | 0.56 | 0.16 | 0.95 |
| TRINITY_DN12437_c0_g1 | Second_Instar | Uncharacterized | 7.05 | 8.45 | 2.39 | 3.49 |
| TRINITY_DN2563_c0_g1 | Second_Instar | similar to secretory Phospholipase A2, partial [*Tribolium castaneum* | 3.96 | 4.12 | 1.17 | 0.95 |
| TRINITY_DN73192_c0_g1 | Second_Instar | zinc finger protein SLUG, putative [Pediculus humanus corporis | 17.09 | 9.12 | 2.62 | 1.65 |
| TRINITY_DN22568_c0_g1 | Second_Instar | engrailed-2 [*Schistocerca gregaria* | 16.51 | 6.74 | 1.96 | 3.56 |
| TRINITY_DN17632_c0_g1 | Second_Instar | Uncharacterized | 15.01 | 5.62 | 1.65 | 6.51 |
| TRINITY_DN19881_c0_g1 | Second_Instar | PREDICTED: protein hairy-like [*Acyrthosiphon pisum* | 23.68 | 14.56 | 3.46 | 0.74 |
| TRINITY_DN11405_c0_g1 | Second_Instar | PREDICTED: DNA mismatch repair protein Mlh1-like [*Acyrthosiphon pisum* | 11.86 | 10.77 | 3.17 | 0.66 |
| TRINITY_DN4719_c0_g6 | Third_Instar | actin-related protein [*Riptortus pedestris* | 8.43 | 0 | 0 | 0 |
| TRINITY_DN17480_c1_g3 | Third_Instar | Uncharacterized | 31.31 | 0 | 22.8 | 0 |
| TRINITY_DN17444_c0_g1 | Third_Instar | PREDICTED: tubulin beta-3 chain-like [*Acyrthosiphon pisum* | 8.3 | 0 | 5.72 | 0.07 |
| TRINITY_DN35297_c1_g3 | Third_Instar | PREDICTED: similar to Rad21 CG17436-PA [*Tribolium castaneum* | 4.01 | 0 | 4.24 | 6.95 |
| TRINITY_DN16471_c0_g1 | Third_Instar | PREDICTED: 39S ribosomal protein L43, mitochondrial-like [*Acyrthosiphon pisum* | 13.79 | 0 | 0 | 0 |
| TRINITY_DN27324_c0_g2 | Third_Instar | PREDICTED: sodium-independent sulfate anion transporter-like isoform X1 [*Apis dorsata* | 3.95 | 0 | 0 | 0 |
| TRINITY_DN10905_c1_g4 | Third_Instar | Uncharacterized | 28.12 | 0 | 0 | 19.99 |
| TRINITY_DN35297_c1_g4 | Third_Instar | PREDICTED: similar to Rad21 CG17436-PA [*Tribolium castaneum* | 2.68 | 0 | 1.22 | 0 |
| TRINITY_DN62034_c0_g2 | Third_Instar | Uncharacterized | 11.2 | 0 | 0 | 0 |
| TRINITY_DN52939_c0_g5 | Third_Instar | actin-related protein [Riptortus pedestris | 18.08 | 0 | 0 | 0 |
| TRINITY_DN25068_c0_g1 | Third_Instar | Uncharacterized | 2 | 0.42 | 0.75 | 6.77 |
| TRINITY_DN18537_c0_g1 | Third_Instar | regulator of G-protein signaling 9-like isoform 1 [*Acyrthosiphon pisum* | 9.8 | 2.29 | 2.82 | 46.33 |
| TRINITY_DN22926_c0_g1 | Third_Instar | Uncharacterized | 90.61 | 21.7 | 35.72 | 101.59 |
| TRINITY_DN19361_c0_g1 | Third_Instar | conserved hypothetical protein [*Pediculus humanus corporis* | 79.93 | 18.61 | 89.17 | 2.25 |
| TRINITY_DN21842_c0_g1 | Third_Instar | hypothetical protein TcasGA2_TC011976 [*Tribolium castaneum* | 9.49 | 2.21 | 4.23 | 59.07 |
| TRINITY_DN14976_c0_g1 | Third_Instar | Uncharacterized | 5.18 | 1.22 | 0.74 | 1.91 |
| TRINITY_DN21233_c0_g1 | Third_Instar | Uncharacterized | 1.88 | 0.44 | 0.76 | 18.87 |
| TRINITY_DN24781_c0_g1 | Third_Instar | Uncharacterized | 5.97 | 1.44 | 3.12 | 4.13 |
| TRINITY_DN18910_c0_g1 | Third_Instar | Uncharacterized | 4.65 | 1.08 | 0.98 | 0.17 |
| TRINITY_DN19469_c0_g1 | Third_Instar | PREDICTED: similar to AGAP004942-PA [*Tribolium castaneum* | 2.03 | 0.46 | 0.71 | 7.27 |
| TRINITY_DN52932_c1_g1 | Adult_Female | nonstructural polyprotein [Aphid lethal paralysis virus | 0 | 0.01 | 1208.14 | 4936.43 |
| TRINITY_DN21076_c3_g9 | Adult_Female | Uncharacterized | 0 | 9952.29 | 22562.03 | 9805.03 |
| TRINITY_DN24270_c2_g1 | Adult_Female | Uncharacterized | 0 | 35.25 | 201.94 | 244.43 |
| TRINITY_DN21076_c3_g5 | Adult_Female | \| hypothetical protein Clole_3223 [Clostridium lentocellum DSM 5427 | 2.06 | 50409.68 | 100243.4 | 38915.53 |
| TRINITY_DN15648_c0_g2 | Adult_Female | hypothetical protein BBA_03436 [*Beauveria bassiana* ARSEF 2860 | 0.13 | 471.68 | 788.91 | 699.73 |
| TRINITY_DN15648_c0_g1 | Adult_Female | hypothetical protein BBA_03436 [Beauveria bassiana ARSEF 2860 | 0.13 | 617.26 | 578.37 | 495.91 |
| TRINITY_DN21076_c3_g4 | Adult_Female | hypothetical protein Clole_3223 [*Clostridium lentocellum* DSM 5427 | 1.24 | 19195.17 | 40283.22 | 17150.45 |
| TRINITY_DN1310_c0_g1 | Adult_Female | Uncharacterized | 0.05 | 1250.06 | 1497.27 | 941.68 |
| TRINITY_DN26599_c0_g1 | Adult_Female | Uncharacterized | 0 | 169.66 | 209.06 | 144.75 |
| TRINITY_DN44711_c0_g1 | Adult_Female | putative antimicrobial knottin protein Btk-4 [*Bemisia tabaci* | 0 | 503.56 | 468.75 | 187.9 |
| TRINITY_DN44226_c0_g1 | Adult_Female | PREDICTED: chromobox protein homolog 5-like isoform 1 [*Nasonia vitripennis* | 18.51 | 18.09 | 34.41 | 53.01 |
| TRINITY_DN20109_c0_g1 | Adult_Female | hypothetical protein YQE_09794, partial [Dendroctonus ponderosae | 1.24 | 2.61 | 2.36 | 3.32 |
| TRINITY_DN22153_c0_g1 | Adult_Female | Uncharacterized | 3.21 | 1.17 | 4.22 | 9.35 |
| TRINITY_DN18851_c0_g1 | Adult_Female | MOF protein [*Danaus plexippus* | 10.59 | 23.59 | 26.6 | 30.41 |
| TRINITY_DN25043_c1_g2 | Adult_Female | Uncharacterized | 0.9 | 0.86 | 2.63 | 2.49 |
| TRINITY_DN6603_c0_g1 | Adult_Female | PREDICTED: trifunctional purine biosynthetic protein adenosine-3 [*Megachile rotundata* | 45.61 | 221.9 | 211.79 | 133.65 |
| TRINITY_DN25137_c0_g2 | Adult_Female | hypothetical protein Kpol_1058p4 [*Vanderwaltozyma polyspora* DSM 70294 | 1.18 | 5.73 | 4.42 | 3.49 |
| TRINITY_DN22551_c0_g1 | Adult_Female | Uncharacterized | 8.36 | 16.28 | 14.28 | 21.94 |
| TRINITY_DN24750_c0_g1 | Adult_Female | Uncharacterized | 15.84 | 7.72 | 15 | 43.83 |
| TRINITY_DN20044_c0_g1 | Adult_Female | PREDICTED: glucose dehydrogenase [acceptor | 7.61 | 20.48 | 24.67 | 22.23 |

Table S7. KEGG-based metabolic pathway annotation for differentially expressed transcripts (DET) for pairwise comparisons between developmental stages of *P. solenopsis* by KOBAS 3.0.

| Compared samples | Kegg Pathways | Total transcripts |
| --- | --- | --- |
|  | Carbon metabolism | 11 |
|  | Pentose and glucuronate interconversions | 2 |
|  | Pyrimidine metabolism | 2 |
|  | MAPK signaling pathway | 2 |
|  | Wnt signaling pathway | 4 |
|  | Ascorbate and aldarate metabolism | 2 |
| EggI vs Second Instar | Drug metabolism - cytochrome P450 | 2 |
|  | Hippo signaling pathway | 2 |
|  | Metabolism of xenobiotics by cytochrome P450 | 2 |
|  | Biosynthesis of amino acids | 3 |
|  | Retinol metabolism | 2 |
|  | FoxO signaling pathway | 7 |
|  | Fatty acid metabolism | 7 |
|  | Amino sugar and nucleotide sugar metabolism | 18 |
|  | Fatty acid biosynthesis | 2 |
|  | Pyrimidine metabolism | 5 |
|  | MAPK signaling pathway | 2 |
| Second Instar vs Third Instar | Wnt signaling pathway | 3 |
|  | Carbon metabolism | 10 |
|  | Glycolysis / Gluconeogenesis | 4 |
|  | Notch signaling pathway | 2 |
|  | Carbon metabolism | 9 |
|  | Amino sugar and nucleotide sugar metabolism | 18 |
|  | Notch signaling pathway | 3 |
|  | Fatty acid biosynthesis | 2 |
| Third Instar vs adult female | Arginine and proline metabolism | 7 |
|  | Glycolysis / Gluconeogenesis | 4 |
|  | Biosynthesis of amino acids | 3 |
|  | FoxO signaling pathway | 5 |
|  | Arachidonic acid metabolism | 2 |
|  | Biosynthesis of amino acids | 3 |
|  | Carbon metabolism | 9 |
| Adult female vs EggI | Fatty acid metabolism | 6 |
|  | Arginine and proline metabolism | 5 |
|  | Oxidative phosphorylation | 2 |
|  | Fatty acid biosynthesis | 2 |

**Table S8**. List of genes associated with biosynthesis of the two major hormone classes sesquiterpenoid juvenile (JH) and the ecdysteroid hormones identified in the *P. Solenopsis* assembled transcriptome at *Maximum Identity* (≥60%) and *E-*value using homologues, particularly from *Acyrthosiphon pisum and Maconellicoccus hirsutus*. FPKM values for each transcript normalized per library are presented. Blue rows represent undetected genes within the *P. Solenopsis* transcriptome for all sequenced stages, while grey rows are those that did not reach the set *Maximum Identity* (≥60%) and *E-*value.

| **GENE** | **BAIT** | **TRANSCRIPTS** | ***E* VALUE** | **Identity %** | **FPKM values normalized per library** | | | | | | |  |
| --- | --- | --- | --- | --- | --- | --- | --- | --- | --- | --- | --- | --- |
|  |  |  |  |  | **Eggs/1^st^ stage** | **2^nd^ stage** | | **3^rd^ stage** | | **adult** | |  |
|  | Juvenile Hormone | | | | | | | | | | |  |
| *Ae-40* | >gi\|193629629\|ref\|XP_001949364.1\| PREDICTED: esterase FE4-like [*Acyrthosiphon pisum* | TRINITY_DN23437_c0_g1  TRINITY_DN20907_c0_g1  TRINITY_DN23566_c0_g1  TRINITY_DN53368_c0_g1  TRINITY_DN23291_c0_g1  TRINITY_DN15588_c0_g1  TRINITY_DN18744_c0_g1  TRINITY_DN22967_c2_g1 | - Low score | Low score |  | - | | - | | -  -  - | |  |
| ***Cyp*** | >gi\|193681039\|ref\|XP_001951093.1\| PREDICTED: probable cytochrome P450 303a1-like [*Acyrthosiphon pisum* | TRINITY_DN22608_c0_g1  TRINITY_DN10885_c0_g1  TRINITY_DN21336_c0_g2  TRINITY_DN23648_c0_g1  TRINITY_DN23648_c0_g1  TRINITY_DN11766_c0_g1  TRINITY_DN14513_c0_g1 | 0  7E-36  0  3E-175  3E-175  2E-167  0 | 79.25  72.82  66.67  62.34  62.34  61.04  60.75 | 46.4  12.26  183.28  13.2  13.2  0.48  7.03 | 16.35  7.07  254.25  6.6  6.6  0.64  7.55 | | 12.37  7.6  267.33  8.88  8.88  1.32  13.7 | | 0.62  22.92  474.51  20.25  20.25  0.28  23.95 | |  |
| ***Cyp450*** | >gi\|328699379\|ref\|XP_001944205.2\| PREDICTED: *cytochrome* P450 4g15-like [*Acyrthosiphon pisum*] | TRINITY_DN57832_c0_g1  TRINITY_DN22077_c0_g1  TRINITY_DN22608_c0_g1  TRINITY_DN10885_c0_g2  TRINITY_DN21253_c0_g1  TRINITY_DN10885_c0_g1  TRINITY_DN22102_c0_g1  TRINITY_DN21336_c0_g2  TRINITY_DN23648_c0_g1  TRINITY_DN23241_c0_g16  TRINITY_DN11766_c0_g1  TRINITY_DN14513_c0_g1 | 4E-56  0  0  8E-110  0  7E-36  0  0  3E-175  7E-86  2E-167  0 | 92.98  81.81  79.25  76.76  74.52  72.82  67.71  66.67  62.34  61.11  61.04  60.75 | 0.98  22.14  46.4  2.51  80.43  12.26  19.85  183.28  13.2  3.94  0.48  7.03 | 0  6.77  16.35  1.1  92.21  7.07  14.91  254.25  6.6  3.12  0.64  7.55 | | 0  7.5  12.37  2.09  103.07  7.6  19.27  267.33  8.88  2.91  1.32  13.7 | | 0  16.25  0.62  4.3  142.87  22.92  212.96  474.51  20.25  73.24  0.28  23.95 | |  |
| ***Jhdk*** | >gi\|124487682\|gb\|ABN11929.1\| juvenile hormone esterase-like protein [Maconellicoccus hirsutus | TRINITY_DN25362_c3_g1  TRINITY_DN21330_c0_g1  TRINITY_DN70739_c0_g1  TRINITY_DN23548_c0_g1  TRINITY_DN23211_c0_g1  TRINITY_DN21442_c1_g1  TRINITY_DN26026_c0_g1  TRINITY_DN24867_c0_g1  TRINITY_DN24900_c0_g1  TRINITY_DN22063_c0_g2  TRINITY_DN19504_c0_g1  TRINITY_DN24506_c2_g2  TRINITY_DN6711_c0_g1  TRINITY_DN24226_c0_g1  TRINITY_DN24506_c0_g2  TRINITY_DN65653_c0_g1  TRINITY_DN19567_c0_g1  TRINITY_DN19567_c1_g1  TRINITY_DN22889_c0_g1  TRINITY_DN66738_c0_g1  TRINITY_DN78760_c0_g1 | 1E-104  4E-102  4E-85  1E-153  0  0  0  2E-142  2E-71  0  2E-88  0  3E-14  0  0  4E-22  3E-122  3E-122  0  1E-22  1E-24 | 100  96.32  92.35  88.96  81.72  81.24  74.86  70.47  68.04  68.01  67.67  67.08  65.85  65.59  65.2  63.41  63.23  61.67  60.77  60.71  60.64 | 8.19  20.18  22.48  12.01  7.94  66.82  7.37  43.13  3.66  0.8  9.04  3.42  12.28  4.2  2.23  1.09  8.12  15.33  1.82  0.85  0 | 3.74  17.12  20.87  3.07  4.05  71.97  7.44  73.89  1.54  0.15  6.75  3.26  33.2  1.47  1.06  0  6.76  22.02  0.48  0  0 | | 4.3  16.88  23.06  5  5.95  72.05  8.5  73.57  2  0.72  10.55  5.26  28.8  1.77  2.57  0  9.89  15.66  0.76  1.12  0 | | 22.41  32.18  29.31  167.34  13  78.4  24.83  101.31  12.97  15.75  11.52  4.71  0  31.89  5  0  12.61  1.35  7.59  1.2  2.11 | |  |
| ***FPS*** | >gi\|307185961\|gb\|EFN71763.1\| Tyrosine-protein kinase Fps85D [*Camponotus floridanus* | TRINITY_DN23401_c0_g3 | 0.00E+00 | 67.68 | 16.04 | 12.8 | | 16.05 | | 21.78 | |  |
| *FPPS2* | gi\|282935089\|gb\|ACT79809.3\| mitochondrial farnesyl diphosphate synthase [*Aphis gossypii* | TRINITY_DN5403_c0_g1  TRINITY_DN14940_c0_g1  TRINITY_DN19105_c0_g1  TRINITY_DN19105_c0_g5  TRINITY_DN19105_c0_g7 | Low score | Low score |  | | - | | - | | -  -  - | |
| *Jhamt* | >gi\|380011984\|ref\|XP_003690071.1\| PREDICTED: malonyl-CoA O-methyltransferase BioC 2-like [*Apis florae* | TRINITY_DN8848_c0_g1  TRINITY_DN15781_c0_g1  TRINITY_DN22034_c0_g1  TRINITY_DN20834_c0_g1  TRINITY_DN18648_c0_g1 | Low score | Low score | - | | - | | - | | -  -  - | |
| ***Jheh*** | >gi\|124487682\|gb\|ABN11929.1\| juvenile hormone esterase-like protein [*Maconellicoccus hirsutus* | TRINITY_DN24867_c0_g1  TRINITY_DN22063_c0_g2  TRINITY_DN24506_c2_g2  TRINITY_DN37988_c0_g1  TRINITY_DN24226_c0_g1  TRINITY_DN24506_c0_g2  TRINITY_DN65653_c0_g1  TRINITY_DN19567_c0_g1  TRINITY_DN19567_c1_g1  TRINITY_DN22889_c0_g1  TRINITY_DN66738_c0_g1  TRINITY_DN78760_c0_g1  TRINITY_DN20903_c0_g2 | 2E-142  0  0  1E-29  0  0  4E-22  3E-122  3E-122  0  1E-22  1E-24  1E-62 | 70.47  68.01  67.08  67.06  65.59  65.2  63.41  63.23  61.67  60.77  60.71  60.64  59.9 | 43.13  0.8  3.42  0  4.2  2.23  1.09  8.12  15.33  1.82  0.85  0  172.43 | | 73.89  0.15  3.26  1.25  1.47  1.06  0  6.76  22.02  0.48  0  0  146.37 | | 73.57  0.72  5.26  0  1.77  2.57  0  9.89  15.66  0.76  1.12  0  122.48 | | 101.31  15.75  4.71  0  31.89  5  0  12.61  1.35  7.59  1.2  2.11  132.99 | |
| ***Jhe_1*** | >gi\|124487682\|gb\|ABN11929.1\| juvenile hormone esterase-like protein [*Maconellicoccus hirsutus* | TRINITY_DN15708_c0_g1  TRINITY_DN24867_c0_g1  TRINITY_DN22063_c0_g2  TRINITY_DN24506_c2_g2  TRINITY_DN6711_c0_g1  TRINITY_DN24226_c0_g1  TRINITY_DN24506_c0_g2  TRINITY_DN65653_c0_g1  TRINITY_DN19567_c0_g1  TRINITY_DN19567_c1_g1  TRINITY_DN22889_c0_g1  TRINITY_DN66738_c0_g1  TRINITY_DN78760_c0_g1 | 2E-15  2E-142  0  0  3E-14  0  0  4E-22  3E-122  3E-122  0  1E-22  1E-24 | 74.51  70.47  68.01  67.08  65.85  65.59  65.2  63.41  63.23  61.67  60.77  60.71  60.64 | 2.73  43.13  0.8  3.42  12.28  4.2  2.23  1.09  8.12  15.33  1.82  0.85  0 | | 1.98  73.89  0.15  3.26  33.2  1.47  1.06  0  6.76  22.02  0.48  0  0 | | 3.29  73.57  0.72  5.26  28.8  1.77  2.57  0  9.89  15.66  0.76  1.12  0 | | 4.02  101.31  15.75  4.71  0  31.89  5  0  12.61  1.35  7.59  1.2  2.11 | |
| ***Jheh_IP5*** | >gi\|193592073\|ref\|XP_001945643.1\| PREDICTED: esterase FE4-like [*Acyrthosiphon pisum* | TRINITY_DN37988_c0_g1  TRINITY_DN65653_c0_g1  TRINITY_DN22889_c0_g1  TRINITY_DN66738_c0_g1  TRINITY_DN20903_c0_g2 | 1E-29  4E-22  0  1E-22  1E-62 | 67.06  63.41  60.77  60.71  59.9 | 0  1.09  1.82  0.85  172.43 | | 1.25  0  0.48  0  146.37 | | 0  0  0.76  1.12  122.48 | | 0  0  7.59  1.2  132.99 | |
| ***Jhebp*** | >gi\|124487682\|gb\|ABN11929.1\| juvenile hormone esterase-like protein [Maconellicoccus hirsutus | TRINITY_DN24867_c1_g5  TRINITY_DN24867_c1_g1  TRINITY_DN24867_c1_g4  TRINITY_DN24867_c1_g2  TRINITY_DN15708_c0_g1  TRINITY_DN7606_c0_g2  TRINITY_DN7606_c0_g1  TRINITY_DN24867_c0_g1  TRINITY_DN22063_c0_g2  TRINITY_DN24506_c2_g2  TRINITY_DN44338_c0_g1  TRINITY_DN6711_c0_g1  TRINITY_DN24226_c0_g1  TRINITY_DN24506_c0_g2  TRINITY_DN65653_c0_g1  TRINITY_DN19567_c0_g1  TRINITY_DN19567_c1_g1  TRINITY_DN22889_c0_g1  TRINITY_DN66738_c0_g1  TRINITY_DN78760_c0_g1 | 6E-22  6E-22  2E-22  2E-21  2E-15  2E-10  2E-10  2E-142  0  0  7E-113  3E-14  0  0  4E-22  3E-122  3E-122  0  1E-22  1E-24 | 77.78  77.78  77.78  76.19  74.51  72.09  72.09  70.47  68.01  67.08  66.31  65.85  65.59  65.2  63.41  63.23  61.67  60.77  60.71  60.64 | 10.95  1.06  4.63  20.07  2.73  0.25  0.98  43.13  0.8  3.42  1.11  12.28  4.2  2.23  1.09  8.12  15.33  1.82  0.85  0 | | 13.82  0.01  12  30.22  1.98  0  2.05  73.89  0.15  3.26  0.35  33.2  1.47  1.06  0  6.76  22.02  0.48  0  0 | | 15.99  1.61  14.23  33.54  3.29  0.54  3.77  73.57  0.72  5.26  0.47  28.8  1.77  2.57  0  9.89  15.66  0.76  1.12  0 | | 2.8  0.11  0.11  20.54  4.02  0  6.86  101.31  15.75  4.71  1.08  0  31.89  5  0  12.61  1.35  7.59  1.2  2.11 | |
| ***Jheh_bp1*** | >gi\|124487682\|gb\|ABN11929.1\| juvenile hormone esterase-like protein [*Maconellicoccus hirsutus* | TRINITY_DN24867_c1_g1  TRINITY_DN24867_c1_g5  TRINITY_DN24867_c1_g4  TRINITY_DN24867_c1_g2  TRINITY_DN15708_c0_g1  TRINITY_DN7606_c0_g2  TRINITY_DN7606_c0_g1  TRINITY_DN24867_c0_g1  TRINITY_DN22063_c0_g2  TRINITY_DN24506_c2_g2  TRINITY_DN6711_c0_g1  TRINITY_DN24226_c0_g1  TRINITY_DN24506_c0_g2  TRINITY_DN65653_c0_g1  TRINITY_DN19567_c0_g1  TRINITY_DN19567_c1_g1  TRINITY_DN22889_c0_g1  TRINITY_DN66738_c0_g1  TRINITY_DN78760_c0_g1 | 6E-22  6E-22  2E-22  2E-21  2E-15  2E-10  2E-10  2E-142  0  0  3E-14  0  0  4E-22  3E-122  3E-122  0  1E-22  1E-24 | 77.78  77.78  77.78  76.19  74.51  72.09  72.09  70.47  68.01  67.08  65.85  65.59  65.2  63.41  63.23  61.67  60.77  60.71  60.64 | 1.06  10.95  4.63  20.07  2.73  0.25  0.98  43.13  0.8  3.42  12.28  4.2  2.23  1.09  8.12  15.33  1.82  0.85  0 | | 0.01  13.82  12  30.22  1.98  0  2.05  73.89  0.15  3.26  33.2  1.47  1.06  0  6.76  22.02  0.48  0  0 | | 1.61  15.99  14.23  33.54  3.29  0.54  3.77  73.57  0.72  5.26  28.8  1.77  2.57  0  9.89  15.66  0.76  1.12  0 | | 0.11  2.8  0.11  20.54  4.02  0  6.86  101.31  15.75  4.71  0  31.89  5  0  12.61  1.35  7.59  1.2  2.11 -  -  - | |
| ***Jheh_Ip2*** | >gi\|76365069\|sp\|Q8MZR6.3\|HYEP1_CTEFE RecName: Full=Juvenile hormone epoxide hydrolase | TRINITY_DN25343_c0_g1  TRINITY_DN22614_c0_g9 | Low score | Low score | - | | - | | - | | -  -  - | |
| ***Jheh_Ip1*** | >gi\|239788286\|dbj\|BAH70830.1\| ACYPI38240 [Acyrthosiphon pisum | TRINITY_DN37988_c0_g1  TRINITY_DN20903_c0_g2 | 1E-29  1E-62 | 67.06  59.9 | 0  172.43 | | 1.25  146.37 | | 0  122.48 | | 0  132.99 | |
| ***Jheh_Ip3*** | >gi\|239788286\|dbj\|BAH70830.1\| ACYPI38240 [Acyrthosiphon pisum | TRINITY_DN37988_c0_g1  TRINITY_DN20903_c0_g2  TRINITY_DN20903_c0_g2 | 1E-29  1.00E-62  1E-62 | 67.06  59.9  59.9 | - | | - | | - | | - | |
| ***Jheh_2*** | >gi\|124487682\|gb\|ABN11929.1\| juvenile hormone esterase-like protein [*Maconellicoccus hirsutus* | TRINITY_DN24867_c0_g1  TRINITY_DN22063_c0_g2  TRINITY_DN24506_c2_g2  TRINITY_DN37988_c0_g1  TRINITY_DN24226_c0_g1  TRINITY_DN24506_c0_g2  TRINITY_DN65653_c0_g1  TRINITY_DN19567_c0_g1  TRINITY_DN19567_c1_g1  TRINITY_DN22889_c0_g1  TRINITY_DN66738_c0_g1  TRINITY_DN78760_c0_g1  TRINITY_DN20903_c0_g2 | 2E-142  0  0  1E-29  0  0  4E-22  3E-122  3E-122  0  1E-22  1E-24  1E-62 | 70.47  68.01  67.08  67.06  65.59  65.2  63.41  63.23  61.67  60.77  60.71  60.64  59.9 | 43.13  0.8  3.42  0  4.2  2.23  1.09  8.12  15.33  1.82  0.85  0  172.43 | | 73.89  0.15  3.26  1.25  1.47  1.06  0  6.76  22.02  0.48  0  0  146.37 | | 73.57  0.72  5.26  0  1.77  2.57  0  9.89  15.66  0.76  1.12  0  122.48 | | 101.31  15.75  4.71  0  31.89  5  0  12.61  1.35  7.59  1.2  2.11  132.99 | |
| *Jheh_Ip4* | >gi\|76365069\|sp\|Q8MZR6.3\|HYEP1_CTEFE RecName: Full=Juvenile hormone epoxide hydrolase | TRINITY_DN25343_c0_g1  TRINITY_DN25343_c0_g1  TRINITY_DN18192_c0_g1  TRINITY_DN18192_c0_g1  TRINITY_DN19140_c1_g1 | Low score | Low score | - | | - | | - | | -  -  - | |
|  | **Ecdysteroid Hormone** | | | | | | | | | | | |
| ***Cyp302a1*** | >gi\|328699379\|ref\|XP_001944205.2\| PREDICTED: cytochrome P450 4g15-like [Acyrthosiphon pisum | TRINITY_DN57832_c0_g1  TRINITY_DN43004_c0_g1  TRINITY_DN22608_c0_g1  TRINITY_DN10885_c0_g2  TRINITY_DN10885_c0_g1  TRINITY_DN21336_c0_g2  TRINITY_DN23648_c0_g1  TRINITY_DN11766_c0_g1  TRINITY_DN14513_c0_g1 | 4E-56  2E-50  0  8E-110  7E-36  0  3E-175  2E-167  0 | 92.98  82.41  79.25  76.76  72.82  66.67  62.34  61.04  60.75 | 0.98  0  46.4  2.51  12.26  183.28  13.2  0.48  7.03 | | 0  1.03  16.35  1.1  7.07  254.25  6.6  0.64  7.55 | | 0  0  12.37  2.09  7.6  267.33  8.88  1.32  13.7 | | 0  0  0.62  4.3  22.92  474.51  20.25  0.28  23.95 | |
| ***Cyp306a1_Aa*** | >gi\|328699379\|ref\|XP_001944205.2\| PREDICTED: cytochrome P450 4g15-like [*Acyrthosiphon pisum* | TRINITY_DN72679_c0_g1  TRINITY_DN57832_c0_g1  TRINITY_DN43004_c0_g1  TRINITY_DN22608_c0_g1  TRINITY_DN10885_c0_g1  TRINITY_DN21336_c0_g2  TRINITY_DN23648_c0_g1  TRINITY_DN21859_c0_g1  TRINITY_DN11766_c0_g1  TRINITY_DN67709_c0_g1  TRINITY_DN14513_c0_g1 | 2E-32  4E-56  2E-50  0  7E-36  0  3E-175  3E-116  2E-167  1E-42  0 | 100  92.98  82.41  79.25  72.82  66.67  62.34  62.05  61.04  60.84  60.75 | 0  0.98  0  46.4  12.26  183.28  13.2  1.4  0.48  4.45  7.03 | | 0  0  1.03  16.35  7.07  254.25  6.6  0.41  0.64  21.18  7.55 | | 0  0  0  12.37  7.6  267.33  8.88  0.64  1.32  16.16  13.7 | | 6.38  0  0  0.62  22.92  474.51  20.25  5.08  0.28  0.35  23.95 | |
| ***Cyp307a1*** | >gi\|124487682\|gb\|ABN11929.1\| juvenile hormone esterase-like protein [*Maconellicoccus hirsutus* | TRINITY_DN72679_c0_g1  TRINITY_DN57832_c0_g1  TRINITY_DN43004_c0_g1  TRINITY_DN22608_c0_g1  TRINITY_DN25561_c0_g1  TRINITY_DN10885_c0_g2  TRINITY_DN10885_c0_g1  TRINITY_DN21336_c0_g2  TRINITY_DN23648_c0_g1  TRINITY_DN11766_c0_g1  TRINITY_DN67709_c0_g1  TRINITY_DN14513_c0_g1  TRINITY_DN15898_c0_g1 | 2E-32  4E-56  2E-50  0  9E-128  8E-110  7E-36  0  3E-175  2E-167  1E-42  0  3E-155 | 100  92.98  82.41  79.25  77.14  76.76  72.82  66.67  62.34  61.04  60.84  60.75  59.16 | 0  0.98  0  46.4  89.53  2.51  12.26  183.28  13.2  0.48  4.45  7.03  6.81 | | 0  0  1.03  16.35  68.36  1.1  7.07  254.25  6.6  0.64  21.18  7.55  5.7 | | 0  0  0  12.37  53.91  2.09  7.6  267.33  8.88  1.32  16.16  13.7  9.74 | | 6.38  0  0  0.62  39.9  4.3  22.92  474.51  20.25  0.28  0.35  23.95  11.42 | |
| ***CYP314A1*** | >gi\|328699379\|ref\|XP_001944205.2\| PREDICTED: cytochrome P450 4g15-like [Acyrthosiphon pisum | TRINITY_DN57832_c0_g1  TRINITY_DN43004_c0_g1  TRINITY_DN70902_c0_g1  TRINITY_DN22608_c0_g1  TRINITY_DN10885_c0_g2  TRINITY_DN10885_c0_g1  TRINITY_DN21336_c0_g2  TRINITY_DN23648_c0_g1  TRINITY_DN11766_c0_g1  TRINITY_DN67709_c0_g1  TRINITY_DN14513_c0_g1 | 4E-56  2E-50  0  0  8E-110  7E-36  0  3E-175  2E-167  1E-42  0 | 92.98  82.41  82.07  79.25  76.76  72.82  66.67  62.34  61.04  60.84  60.75 | 0.98  0  82.71  46.4  2.51  12.26  183.28  13.2  0.48  4.45  7.03 | | 0  1.03  87.85  16.35  1.1  7.07  254.25  6.6  0.64  21.18  7.55 | | 0  0  82.89  12.37  2.09  7.6  267.33  8.88  1.32  16.16  13.7 | | 0  0  141.05  0.62  4.3  22.92  474.51  20.25  0.28  0.35  23.95 | |
| ***Cyp315a1*** | >gi\|328699379\|ref\|XP_001944205.2\| PREDICTED: cytochrome P450 4g15-like [*Acyrthosiphon pisum* | TRINITY_DN57832_c0_g1  TRINITY_DN43004_c0_g1  TRINITY_DN22608_c0_g1  TRINITY_DN10885_c0_g2  TRINITY_DN10885_c0_g1  TRINITY_DN21336_c0_g2  TRINITY_DN23648_c0_g1  TRINITY_DN11766_c0_g1  TRINITY_DN14513_c0_g1 | 4E-56  2E-50  0  8E-110  7E-36  0  3E-175  2E-167  0 | 92.98  82.41  79.25  76.76  72.82  66.67  62.34  61.04  60.75 | 0.98  0  46.4  2.51  12.26  183.28  13.2  0.48  7.03 | | 0  1.03  16.35  1.1  7.07  254.25  6.6  0.64  7.55 | | 0  0  12.37  2.09  7.6  267.33  8.88  1.32  13.7 | | 0  0  0.62  4.3  22.92  474.51  20.25  0.28  23.95 | |
| ***Cyp18a1*** | >gi\|328699379\|ref\|XP_001944205.2\| PREDICTED: cytochrome P450 4g15-like [*Acyrthosiphon pisum* | TRINITY_DN57832_c0_g1  TRINITY_DN43004_c0_g1  TRINITY_DN22608_c0_g1  TRINITY_DN10885_c0_g1  TRINITY_DN24739_c0_g1  TRINITY_DN21336_c0_g2  TRINITY_DN7147_c0_g2  TRINITY_DN7147_c0_g1  TRINITY_DN23648_c0_g1  TRINITY_DN20414_c0_g1  TRINITY_DN22082_c0_g1  TRINITY_DN11766_c0_g1  TRINITY_DN67709_c0_g1  TRINITY_DN14513_c0_g1 | 4E-56  2E-50  0  7E-36  1E-59  0  0  0  3E-175  5E-100  3E-169  2E-167  1E-42  0 | 92.98  82.41  79.25  72.82  72.68  66.67  64.83  64.83  62.34  61.59  61.34  61.04  60.84  60.75 | 0.98  0  46.4  12.26  10.43  183.28  16.88  7.15  13.2  10.82  11.23  0.48  4.45  7.03 | | 0  1.03  16.35  7.07  7.51  254.25  19.12  7.13  6.6  15.35  12.25  0.64  21.18  7.55 | | 0  0  12.37  7.6  6.89  267.33  19.34  7.36  8.88  24.28  11.38  1.32  16.16  13.7 | | 0  0  0.62  22.92  7.76  474.51  9.93  12.08  20.25  0.63  9.78  0.28  0.35  23.95 | |
| ***3-Dehydroecdysone-3-alpha-reductase*** | >gi\|89473706\|gb\|ABD72665.1\| putative diacetyl/L-xylulose reductase [*Acyrthosiphon pisum* | TRINITY_DN62215_c0_g1  TRINITY_DN45337_c0_g1  TRINITY_DN52943_c0_g1  TRINITY_DN63054_c0_g1  TRINITY_DN8715_c0_g2  TRINITY_DN50428_c0_g1  TRINITY_DN8715_c0_g1  TRINITY_DN13998_c0_g1  TRINITY_DN11277_c0_g1  TRINITY_DN16411_c0_g1  TRINITY_DN17342_c0_g1  TRINITY_DN21516_c0_g1  TRINITY_DN24530_c0_g1  TRINITY_DN25696_c3_g2  TRINITY_DN35238_c0_g1  TRINITY_DN16238_c0_g1  TRINITY_DN2656_c0_g2  TRINITY_DN2656_c0_g1  TRINITY_DN17292_c0_g1  TRINITY_DN16750_c0_g1 | 2E-150  2E-34  2.00E-35  0  5E-106  1E-34  1E-105  1E-43  3E-105  0  7E-149  4E-103  1E-109  5E-110  1E-81  3E-178  2E-82  2E-82  5E-72  1E-79 | 100  100  100  99.71  98.98  98.65  98.47  83.96  67.42  65.61  65.46  64.62  64.22  64.17  63.41  63.02  61.75  61.75  61.23  59.93 | 0  0  0  0.66  1.58  1.1  42.42  71.13  107.26  35.08  30.59  84.1  73.56  54.44  126.79  14.73  21.16  75.4  33.54 | | 0  0  0  0.06  0  0.06  45.45  46.19  84.51  31.31  12.98  60.52  56.86  64.26  158.26  21.14  26.21  80.93  36.4 | | 0  0  0  0  0  0  51.02  46.52  112.1  29.72  9.08  56.18  58.2  84.64  183.62  15.19  31.17  118.93  36.34 | | 7.43  6.24  4.04  0  0  0  45.84  122.5  80.3  23.76  6.77  74.4  77.42  52.67  232.08  12.08  22.55  95.05  33.38 | |
| *nvd* | >gi\|512888352\|ref\|XP_004922073.1\| PREDICTED: methenyltetrahydrofolate synthase domain-containing protein-like [*Bombyx mori* | TRINITY_DN24074_c0_g1  TRINITY_DN22509_c0_g2 | Low score | Low score | - | | - | | - | | -  -  - | |
| ***Ecdysone_Oxidase*** | >gi\|91085207\|ref\|XP_972126.1\| PREDICTED: similar to alcohol dehydrogenase [*Tribolium castaneum* | TRINITY_DN22505_c0_g3  TRINITY_DN22505_c0_g2  TRINITY_DN44118_c0_g1  TRINITY_DN24626_c0_g2  TRINITY_DN21049_c0_g1  TRINITY_DN22783_c0_g1  TRINITY_DN22505_c0_g1  TRINITY_DN24515_c0_g1  TRINITY_DN11750_c0_g1  TRINITY_DN24393_c0_g1  TRINITY_DN62011_c0_g1  TRINITY_DN14552_c0_g2  TRINITY_DN14552_c0_g1  TRINITY_DN23095_c0_g1  TRINITY_DN24989_c0_g1 | 3E-27  9E-29  1E-23  0  0  0  0  0  1E-14  0  6E-126  0  0  0  0 | 92.19  91.04  82.09  77.65  75.65  75.47  73.02  72.49  69.49  63.9  62.28  60.96  60.96  60.94  59.9 | 0  0  1.3  7.7  56.62  20.24  7.2  15.73  2.73  4.98  0.74  4.42  0  1.72  2.6 | | 0  0  0.41  2.71  61.74  6.74  3.12  5.6  14.31  1.8  0.08  5.26  0.75  1.44  1.87 | | 0  0  0  1.88  72.98  5.25  5.43  7.6  29.1  2.4  0.31  3.96  1.59  2.94  3.15 | | 0  0  0.86  2.44  95.63  1.18  0.67  12.27  7.16  6.1  0.15  0.06  0.06  3.99  8.94 | |
